# Intermediate-range solvent templating and counterion behaviour at charged carbon nanotube surfaces

**DOI:** 10.1038/s41565-025-01865-9

**Published:** 2025-02-21

**Authors:** Camilla Di Mino, Thomas F. Headen, Nadir S. Basma, David J. Buckley, Patrick L. Cullen, Martin C. Wilding, Milo S. P. Shaffer, Neal T. Skipper, Adam J. Clancy, Christopher A. Howard

**Affiliations:** 1 Department of Physics and Astronomy, University College London, London, UK; 2 Department of Materials, University of Oxford, Oxford, UK; 3 ISIS Neutron and Muon Source, Science and Technology Facilities Council, Rutherford Appleton Laboratory, Didcot, UK; 4 Molecular Sciences Research Hub, Department of Chemistry, Imperial College London, London, UK; 5 School of Engineering and Materials Science, Queen Mary University of London, London, UK; 6 UK Catalysis Hub, Rutherford Appleton Laboratory, Didcot, UK; 7 Department of Materials Science, Imperial College London, London, UK; 8 Department of Chemistry, University College London, London, UK

**Keywords:** Nanoscience and technology, Materials science

## Abstract

The ordering of ions and solvent molecules around nanostructures is of profound fundamental importance, from understanding biological processes to the manipulation of nanomaterials to optimizing electrochemical devices. Classical models commonly used to describe these systems treat the solvent simplistically, an approach that endures, in part, due to the extreme difficulty of attaining experimental measurements that challenge this approximation. Here we perform total neutron scattering experiments on model systems—concentrated amide solutions of negatively charged carbon nanotubes and sodium counterions—and measure remarkably complex intermediate-range molecular solvent ordering. The charged surface orders the solvents up to ∼40 Å, even beyond its dense concentric solvation shells. Notably, the molecular orientation of solvent in direct contact with the nanotube surface itself is distinct, lying near-parallel and not interacting with desolvated sodium counterions. In contrast, beyond this layer the ordering of solvent is perpendicular to the surface. Our results underscore the critical importance of multibody interactions in solvated nanoscale systems and charged surfaces, highlighting competing ion/surface solvation effects.

## Abstract (web summary)

Neutron scattering on a model system of highly concentrated solutions of charged carbon nanotubes reveals a strong solvent ordering up to ∼40 Å around the charged nanoscale surface.

## Main

Nanoscale species dispersed in liquid media are ubiquitous across the physical, material and biological sciences. Well-known examples include dyes, asphaltenes, liquid crystals^1^, proteins^2^, nucleic acids^3^, clays, viruses^4^, polymers, composites^5^ and nanomaterials^6^. For biological molecules, local solvent coordination can determine their three-dimensional structure and functionality^2^. For functional nanomaterials, the creation of liquid dispersions enables the processing of many current and envisaged technologies via printing, casting or embedding into films, composites and devices in large volume at low cost^7^. Despite the clear importance of understanding and controlling these systems, due to their inherent multi-length-scale complexity, the most common approach is to apply classical continuum models, such as Derjaguin–Landau–Verwey–Overbeek theory^8^, which have proved relatively successful at describing dispersions of >50 nm particles. However, as reviewed by Batista et al.^9^, in many cases the behaviour of nanoparticle dispersions deviates substantially from the predictions of these models, particularly when the nanoparticle dimension(s) approach 1–20 nm. The failure of these theories is attributed to the breakdown of certain classical assumptions at the nanoscale. Most notable is the simplistic treatment of the solvent as a uniform-density dielectric continuum with polarity modelled by a single parameter, that is, the relative permittivity, *ε*_r_, ignoring molecular shape and orientation. In fact, far from representing a homogeneous field, the local arrangement of individual solvent molecules around the nanoparticle can vary substantially, interdependently determined by local effects including intersolvent bonding, steric effects, local charge anisotropy and proximity to surfaces and/or charged species^9,10^. In contrast, for simple molecular/atomic solutes, the three-dimensional (3D) ordering of solvent molecules into well-defined solvation shells is well understood to lower the free energy of the system, resulting in thermodynamically favourable dissolution. The effects of solvent ordering become increasingly important as the solvent molecules become comparable in size to one or more dimensions of the solute species^9,11^. Electrolyte ordering at nanostructured (charged) surfaces also underpins the performance of many electrochemical devices, most directly for supercapacitors. However, to date, the focus has been on ion distribution^12–16^, with solvent ordering considered to be of secondary importance, and either ignored or modelled by a surface-adjacent modified dielectric region, required to account for failures of uniform-density dielectric models^17–19^.

Experimental determination of the solvent ordering at surfaces or around nanosized species is particularly challenging due to the small fraction of solvent at the interface. Diffraction experiments, which measure the average structure of the whole system, suffer from the typically low concentration of the relevant correlations and the heterogeneous nature of the dispersed particles or surfaces. Nevertheless, X-ray diffraction has been applied to determine solvent density fluctuations around dispersed nanoparticles^20,21^ and, by exploiting the power of data-constrained modelling, has provided evidence for the partial desolvation of ions within microporous supercapacitor electrodes^14^. However, X-rays are only weakly sensitive to hydrogen atoms, the most abundant component of most solvents, and thus to the orientation of solvent molecules around ions or surfaces^20^, and the use of a single dataset can lead to multiple viable and consistent interpretations^21,22^. Far greater detail can be provided by neutron scattering because neutrons are strongly scattered by hydrogen. Moreover, as neutron scattering lengths differ in sign between protons and deuterons, measuring systems with isotopically distinct solvents provides multiple scattering profiles without modifying the system structure. By combining multiple diffraction datasets and encoding constraints, such as internal molecular structure and intermolecular overlap^23^, into the analysis, neutron diffraction can elucidate solvent ordering about molecules/ions in solution^24–26^ in unparalleled detail.

Single-walled carbon nanotubes (SWCNTs) present an ideal platform to study nanoparticle solvation. Their uniformly small diameter (∼1 nm) and near-perfect cylindrical shape provide a simple model compared to faceted crystalline nanoparticles with a heterogeneous distribution of sizes and shapes^9^. Furthermore, SWCNTs are highly soluble (>10 mg ml^−1^) in aprotic polar solvents when they are negatively charged^7,27–29^. High concentrations are required to increase the fraction of surface-adjacent correlations to influence the total scattering profile. Finally, the ordering of solvent molecules and ions around charged SWCNTs is particularly relevant for understanding electrochemical devices because many electrodes (most notably for electrochemical double-layer supercapacitors and lithium-ion batteries) consist of charged *sp*^2^-hybridized carbons.

**Fig. 1:**
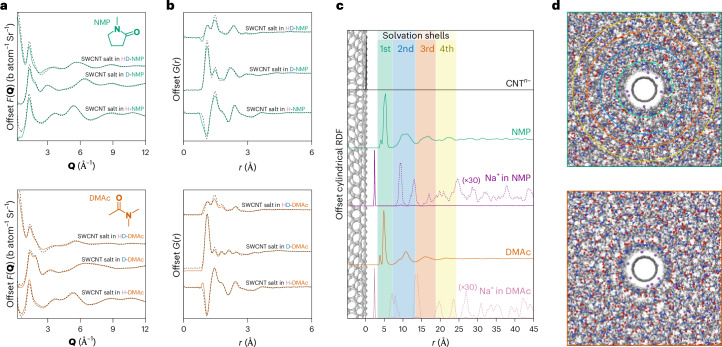
Neutron diffraction and EPSR-derived structures of concentrated solutions of charged SWCNTs in NMP and DMAc. **a**, Total structure factors obtained from neutron diffraction measurements of SWCNT solutions in NMP and DMAc (black dashed lines) in barns (b) per atom per steradian (Sr), and fit from the EPSR-refined simulation (green/orange solid lines), offset for clarity. Larger-scale *F*(**Q**) plots are provided in Supplementary Figs. 3 and 4. **b**, Composite RDFs for NMP and DMAc, obtained by Fourier transformation of the structure factors, offset for clarity. Larger-scale *G*(*r*) plots are provided in Extended Data Figs. 1 and 2. **c**, cRDFs from the SWCNT central axis to the CoM of species, normalized (0,1) and offset for clarity. From top to bottom: SWCNT carbons (black), NMP CoM (green), sodium ions in NMP NaSWCNT solution (purple, ×30 dashed), DMAc CoM (orange), sodium ions in DMAc NaSWCNT solution (pink, ×30 dashed). Shaded backgrounds correspond to the solvation shell of NMP. Solvation shell boundaries are defined by minima at (NMP) 5.9, 12.1, 17.1 and 21.9 Å and (DMAc) 6.1, 11.9, 17.0 and 21.9 Å from the SWCNT surface. Solvation shell density maxima are found at (NMP) 4.7, 9.8, 15.0 and 20.0 Å and (DMAc) 4.3, 9.7, 14.4 and 19.4 Å. Ammonia cRDF and structure are discussed in Supplementary Information 2. **d**, Ball-and-stick molecular graphics snapshots of the structure around SWCNTs of NMP solution (top) and DMAc solution (bottom), derived from neutron-diffraction-refined EPSR (SWCNT, black; non-SWCNT carbon, grey; oxygen, orange; nitrogen, blue; hydrogen, white; sodium, purple), with overlaid solvation shell outer radii in the NMP structure. Shell radii are omitted for DMAc to better highlight the cylindrical arrangement of solvent molecules.

**Extended Data Fig. 1:**
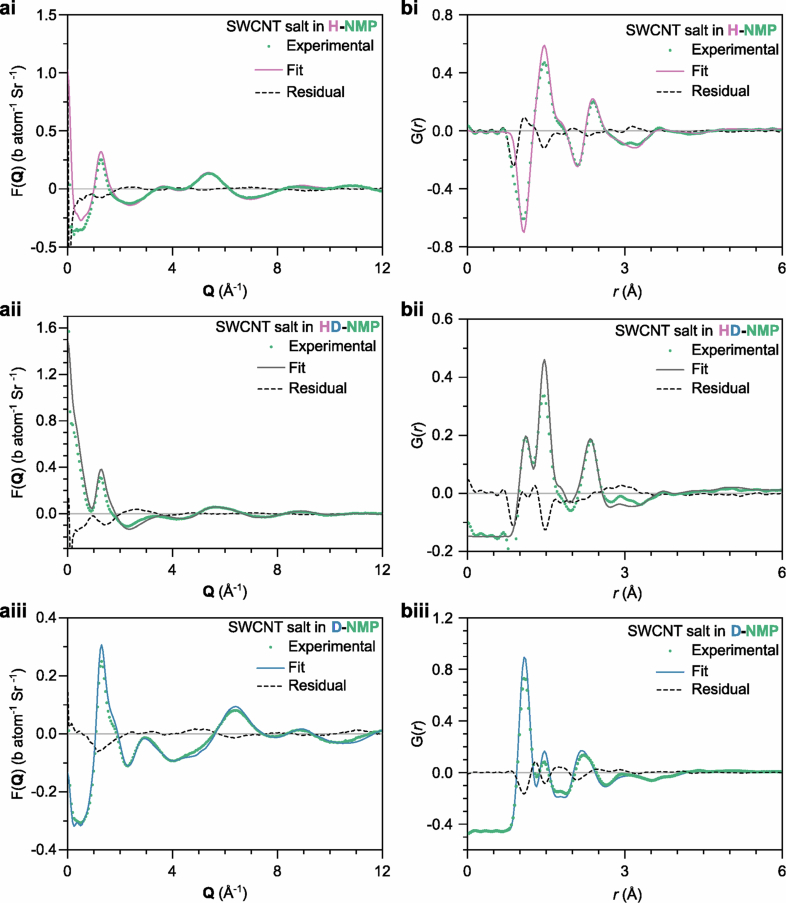
Higher magnification neutron data and EPSR fits (with residual) of NMP solutions of nanotubide salt. (**a**) Total structure factors and (**b**) Composite radial distribution functions, obtained by Fourier transformation of the structure factors of 25 mg mL^−1^ nanotubide solution in different isotopic contrasts of NMP. (i) H-NMP, C_5_H_9_NO. (ii) 50:50 molar mixture of H-NMP and D-NMP, (iii) D-NMP, C_5_D_9_NO. Green circles – Experimentally acquired data and Fourier Transform thereof; Solid line – Data from EPSR-refined simulation; Black dashed line – Residual (Fit-Experimental). Guide to the eye at y = 0 provided as pale grey line for comparison to residual.

**Extended Data Fig. 2:**
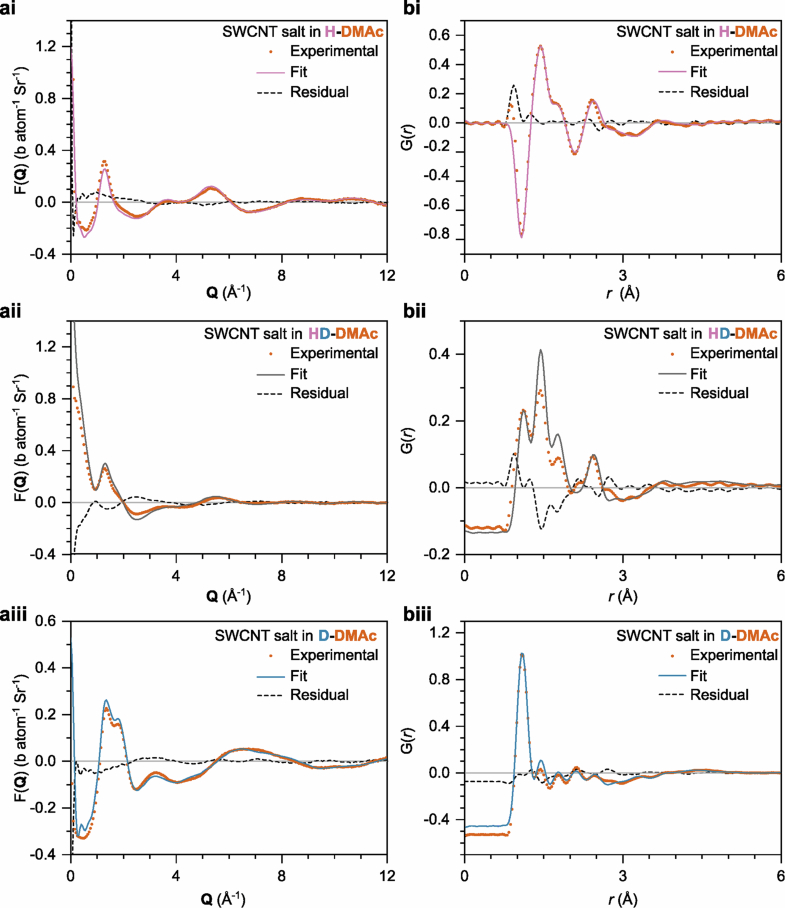
Higher magnification neutron data and EPSR fits (with residual) of DMAc solutions of nanotubide salt. (**a**) Total structure factors and (**b**) Composite radial distribution functions, obtained by Fourier transformation of the structure factors of 25 mg mL^-1^ nanotubide solution in different isotopic contrasts of DMAc. (i) H-DMAc, C_4_H_9_NO. (ii) 50:50 molar mixture of H-DMAc and D-DMAc, (iii) D-DMAc, C_4_D_9_NO. Vermillion circles – Experimentally acquired data and Fourier Transform thereof; Solid line – Data from EPSR-refined simulation; Black dashed line – Residual (Fit-Experimental). Guide to the eye at y = 0 provided as pale grey line for comparison to residual.

Here, concentrated solutions of SWCNT anions and sodium cations were formed by dissolving the ionic nanotube salt in two polar aprotic solvents (25 mg_NaSWCNT_ ml_(solvent)_^−1^). We selected suitable solvents of different molecular geometry, namely, *N*,*N*-dimethylacetamide (DMAc) and *N*-methylpyrrolidone (NMP), and used three isotopically distinct mixtures for each solvent (protiated, deuterated, 50:50). We note that the syntheses of SWCNTs and NaSWCNT introduce a small fraction of iron and ammonia, respectively, which are discussed in Supplementary Discussion 2. Solutions were measured using total neutron scattering with detailed 3D modelling-enhanced analysis performed via empirical potential structure refinement^30^ (EPSR) (Supplementary Information 1). In summary, this method uses a classical Monte Carlo simulation of the system, starting with standard ‘reference’ interatomic potentials (Lennard–Jones and Coulomb) (Supplementary Table 1); an additional ‘empirical potential’ is then derived from the mismatch between the simulated and experimental scattering, thus driving the simulation to match all the available datasets. This iterative process results in a spatially and orientationally resolved atomistic model of the system, consistent with the available scattering data. Experimental total structure factors (*F*(**Q**)) are presented in Fig. 1a (and Extended Data Figs. 1 and 2) alongside the EPSR-calculated *F*(**Q**), which closely match the experimental data across the full **Q**-range for all three isotopic substitutions. Direct Fourier transformation of the *F*(**Q**)s yields the real-space, composite total radial distribution functions (RDFs), *G*(*r*)s (Fig. 1b).

## Intermediate-range solvent ordering from the nanointerface

To examine the solvation of the anionic SWCNTs, in Fig. 1c we plot the cylindrical radial distribution functions^31^ (cRDFs) of each solvent’s centre-of-mass (CoM) calculated outwards from the nanotube central axis and offset by the modelled SWCNT radius. Around the SWCNT, it is immediately clear that the solvent structure is heavily disrupted, and the data reveal four distinct solvation shells organized as concentric cylinders around the SWCNT. Approximately 20% of all the DMAc and NMP molecules in the system are found within these solvation shells (within ∼25 Å of the SWCNT; Supplementary Tables 2 and 3). The average distances between subsequent solvation shells are similar to the inter-amide self-solvation distances in the pure solvent^26,32^. The first solvation shell is almost double the density of bulk solvent, with the subsequent shells having lower density in compensation.

**Fig. 2:**
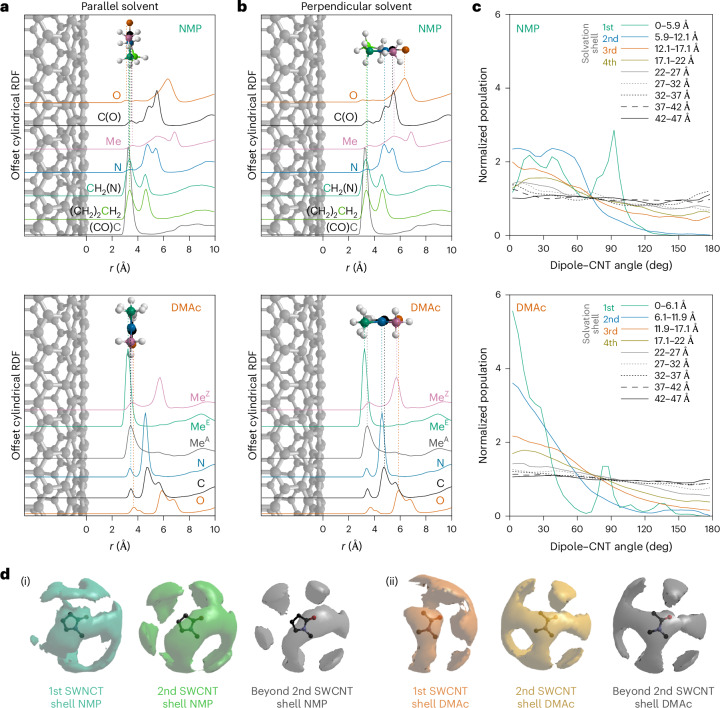
The orientational structure of NMP and DMAc around SWCNTs and intersolvent structure. **a**,**b**, cRDFs of constituent solvent atoms from the SWCNT surface, overlaid with schematic orientations of solvents versus nanotube surface for a monolayer of solvent molecules closest to the SWCNT, as seen in partial *g*(*r*) as small peaks at equivalent distances (**a**), and for solvents at further distances with dipole-ordered orientation with oxygen the furthest atom from the SWCNT, and CH_*n*_ species closest to the SWCNT surface (**b**). Species in **b** stack with the molecular plane perpendicular to the solvent–SWCNT vector as seen in the distribution of orientational histograms (Extended Data Fig. 5). Atom/line colours: O, orange; N, blue; carbonyl C, black; H, white. NMP: N-methyl C, pink; C3, grey; C4, green; C5, blue-green. DMAc: acetic C, grey; E-methyl C, green; Z-methyl C, pink. **c**, Distribution of orientations of the angle between the amide dipole vector and a (radial) vector pointing away from the nanotube central axis, normalized with 1 as an even distribution across all angles. Distances from the SWCNT surface are binned by solvation shells and at 5 Å intervals at further distances. **d**, Amide–amide SDF for solvent molecules in the first SWCNT solvation shell, the second SWNCT solvation shell, and beyond the second SWCNT solvation shell (>12.2 Å and >11.9 Å from the SWNCT wall for NMP and DMAc, respectively), for NMP and DMAc. The amide–amide SDF of all solvents is provided in Extended Data Fig. 6.

**Extended Data Fig. 3:**
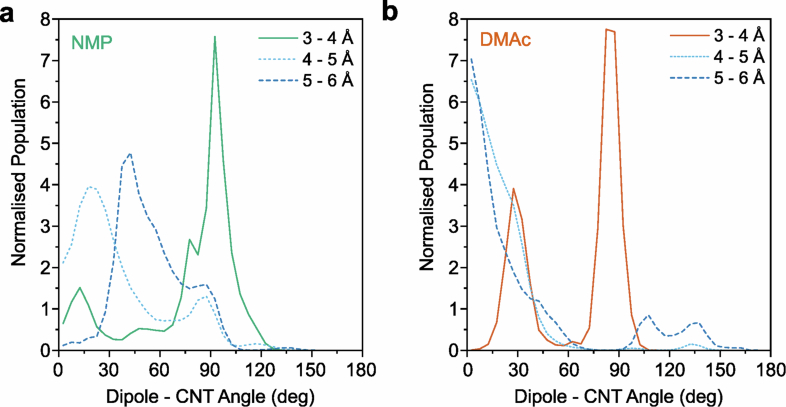
Angular radial distribution functions of solvent molecules closest to SWCNT surface. Distribution of orientations of the angle between amide dipole vector and vector pointing towards the nanotube central axis for NMP (**a**) and DMAc (**b**). Distances from SWCNT surface provide in 1 Å bins within the first solvation shell, showing how ~90° orientation of solvents (that is, dipole parallel to nanotube axis) occurs for the closest solvent molecules, while further molecules within the first solvation shell (4- 6 Å) have dipoles directed towards the nanotube (<90°).

To investigate how the amide molecules are arranged within the solvation shells, we plot the cRDFs between the SWNCT and each solvent atom type (Fig. 2a) and cylindrical orientational distribution functions (CODFs) of the relative orientation of the solvent molecule dipole normal to the SWCNT surface (Fig. 2c). The affected solvent molecules preferentially align according to two distinct orientations. Within the first solvation shell, a fraction of the closest layer of molecules (Fig. 1c; for NMP, 3.6 Å; for DMAc, 3.4 Å) lie parallel to the nanotube, maximizing the interaction between the surface and the resonant O=C–N amidic backbone. This configuration is reflected in a peak at ∼90° in the CNT-dipole CODFs, in a distance range <6.1 Å from the nanotube surface, and it is confirmed by the partial cRDFs (Fig. 2c) where weak equidistant peaks appear at ∼4 Å for all backbone atoms (Fig. 2a). Narrower binning of CODF distances in the first solvation shell confirms that this effect is most prominent at the closest distances (Extended Data Fig. 3).

**Extended Data Fig. 4:**
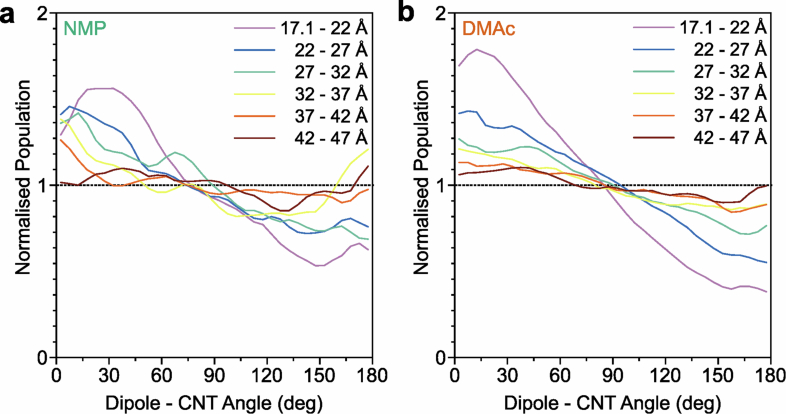
Angular radial distribution functions of solvent molecules beyond the first three solvation shells. Distribution of orientations of the angle between amide dipole vector and vector pointing towards the nanotube central axis for NMP (**a**) and DMAc (**b**). Distances from SWCNT surface binned to highlight 4^th^ solvation shell (17.1–22 Å) and 5 Å bins thereafter. Random population (y = 1) denoted by dashed black line, highlighting preferential dipole alignment (<90°) beyond the solvation shells.

**Extended Data Fig. 5:**
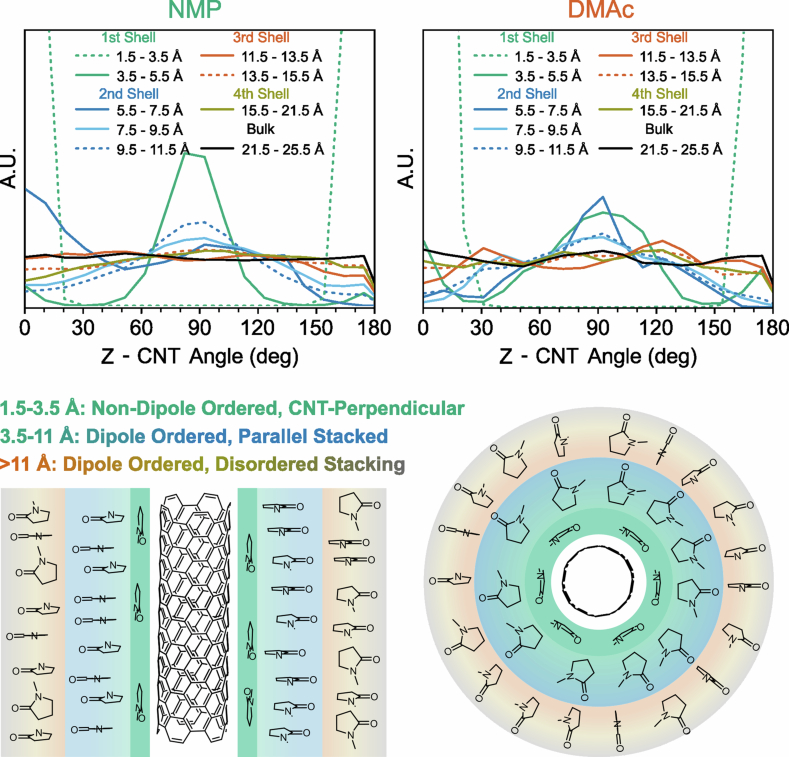
Out-of-plane solvent ordering aRDFs. Distribution of orientations of the angle between amide z vector (normal to molecular plane) and vector pointing towards the nanotube central axis. Distances from SWCNT surface binned by 2 Å intervals within first 3 solvation shell regimes (colour coordination used to distinguish general distinction between shells) and 4 Å bins for 4^th^ shell and beyond. (Left) NMP, (Right) DMAc; (Bottom) Schematic of solvent ordering regimes as function of distance from the SWCNT wall normal and down the SWCNT axis, respectively. NMP selected to better highlight molecular plane stacking. Not to scale.

At further distances (including the majority of the first solvation shell), there is a preference for the amides to orient with their dipole moment directly away from the negatively charged SWCNT surface (Fig. 2c), and the degree of this molecular ordering decreases as a function of distance. Remarkably, the CODFs show that the orientation of the amide molecules is influenced by the SWCNT up to over 40 Å from the SWCNT wall for both solvents. Only beyond this distance, as we approach half the average experimental nanotube separation, is the effect of the nanotube on the solvent molecules’ average orientation not discernible (Extended Data Fig. 4). The orientation bias seen beyond the solvation shells appears sufficiently small as to not substantially disrupt average intersolvent distances. Within the first two solvation shells, the dipole-ordered solvents (≳4 Å from the SWCNT surface) also have an intersolvent stacking order influenced by the nanotube, with their molecular planes preferentially lying flat, to stack along the nanotube (Extended Data Fig. 5). Beyond, while maintaining dipole ordering, the molecules are free to rotate about the dipole with no stacking order seen. The non-dipole-ordered closest solvent molecules within the first solvation shell (≲4 Å) also show distinct ordering of their molecular planes, lying flat against the nanotube, forming C–H···SWCNT^*n*−^ interactions.

## Solvent–solvent structure in (and beyond) solvation shells

**Fig. 3:**
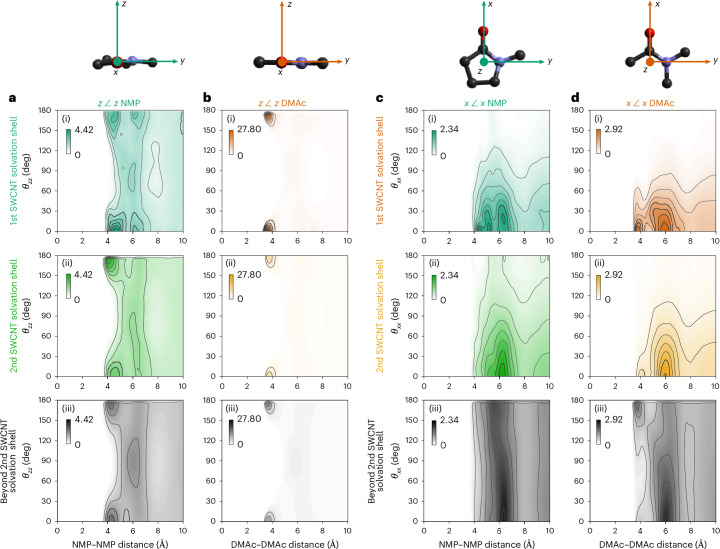
Solvent–solvent relative orientation as a function of CoM–CoM distance for solvents in the first SWCNT solvation shell, the second SWCNT solvation shell and beyond the second SWCNT solvation shell. **a**–**d**, Angular RDFs of NMP (**a**,**c**) and DMAc (**b**,**d**) with defined axes (top) of *x* along the C=O axis that approximates the direction of the dipole and *z* normal to the molecular plane. Inter-*z* (*θ*_*zz*_, **a**,**b**) and inter-*x* (*θ*_*xx*_, **c**,**d**) angles, binned in 10° increments. Data sampled from solvents with CoM found within (i) the first SWCNT solvation shell (NMP-SWCNT < 5.9 Å; DMAc-SWCNT < 6.1 Å), (ii) the second SWCNT solvation shell (NMP-SWCNT 5.9–12.1 Å; DMAc-SWCNT 6.1–11.9 Å) and (iii) beyond the second SWCNT solvation shell (>12.2 Å and >11.9 Å from the SWNCT wall for NMP and DMAc, respectively). The *g*(*r*,*θ*) intensity is given by colour scale inset in each graph, and is scaled with the maximum set to the highest intensity *g*(*r*) of a given solvent-axes set. The aRDFs of all solvent molecules in the analysis cell are provided in Extended Data Fig. 6, and data scaled to the *g*(*r*) range of each individual aRDF are provided in Extended Data Fig. 7.

The intersolvent (DMAc:DMAc and NMP:NMP) spatial arrangement within the first SWCNT solvation shell also differs from that of the rest of the solution. This arrangement can be examined by plotting 3D amide–amide spatial density functions (SDFs, Fig. 2d), which show the most likely positions of neighbouring solvent molecules, and the amide–amide angular RDFs (aRDFs, Fig. 3), which show preferential angles between adjacent amide molecular planes (*z* axes, *θ*_*zz*_) and adjacent amide dipoles (*x* axes, *θ*_*xx*_). Here, we group together solvents by distance from the SWCNT, within the first (Fig. 3a(i),b(i),c(i),d(i)) and second (Fig. 3a(ii),b(ii),c(ii),d(ii)) solvation shells, and beyond the second SWCNT solvation shell (Fig. 3a(iii),b(iii),c(iii),d(iii)). Each solvent selection is then plotted as intersolvent CoM–CoM distance versus the angle between either normal to the molecular plane (*z* axis, Fig. 3a,b) or solvent dipoles (*x* axis, Fig. 3c,d). For amide molecules in the surface monolayer, adjacent solvents stack near-planar (*θ*_*zz*_ ≈ 0/180°, Fig. 3a(i),b(i)) with near-parallel dipoles (*θ*_*xx*_ ≈ 0°, Fig. 3c(i),d(i)), forming a ring around the SWCNT.

The nanotube also occludes one side of the immediately adjacent amide molecules. In DMAc, the SDF of the first solvation shell shows little density of adjacent solvent around the amidic and *trans*-oxygen methyl groups (Fig. 2d). Instead, high solvent density is seen as discrete lobes both above/below the molecular plane, and in-plane by the oxygen. For NMP, the case is less defined due to the presence of multiple similar hydrogen environments, but there is clearly still a level of occlusion from the nanotube near all three CH_2_ sites (Fig. 2d). This behaviour is absent for both within and beyond the second solvation shell, which share the same SDF features.

Planar stacking is still preferred in the second shell, albeit with less defined *g*(*r*,*θ*_*zz*_) features (Fig. 3a(ii),b(ii)). The DMAc–DMAc stacking passes from parallel, in the region close to the negative surface, to antiparallel at further distances (Fig. 3d(iii)), reminiscent of the structure of pure liquid DMAc, whereas no stacking ordering is seen for NMP–NMP (Fig. 3c(iii)) in the bulk^26,32^. The non-templated structure of NMP both in the pure liquid and in these high-concentration solutions is potentially linked to NMP’s ability to disperse carbon nanomaterials because there is little energetic cost from disrupting the self-solvation structure^33^. In fact, despite the reorientation of amide molecules directed by the SWCNT, it is interesting to note that the average intersolvent arrangement, that is, within and across the solvation shells, is relatively unperturbed (Fig. 3). Thus, the structure of these liquid amides is largely maintained despite the solvation of high concentrations of dissolved SWCNTs, consistent with the small measured differences in their respective *F*(**Q**)s (Supplementary Fig. 7).

## Sodium counterion distribution and solvation

The densely packed and intricately ordered solvent molecules around the SWCNTs markedly contrast with the uniform description of the solvent in many models used to describe (nano)particle dispersions^7,9,34^. Another key tenet of these models is the formation of a diffuse electrical double layer. In most theories, the counterions exist in two distinct states: condensed on the charged surface as an internal Stern layer, or dissociated from the nanoparticle/surface, forming a diffuse Gouy–Chapman layer with concentration diminishing exponentially from a maximum adjacent to the Stern layer. The charge from the partially screened surface is theorized to lead to inter-nanomaterial repulsion (often considered additively to counter van der Waals attractions) as a rationale for nanoparticle dispersion stability^9^.

**Fig. 4:**
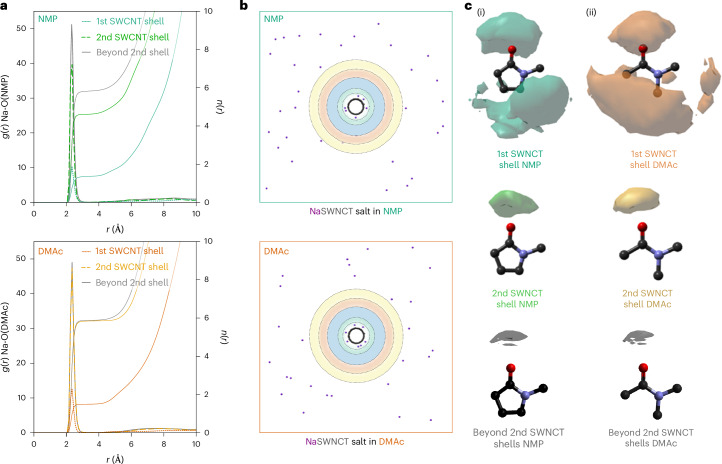
Distribution and solvation of the sodium counterions. **a**, RDF and cumulative coordination number of the solvent geometric centre of sodium for solvents in the first SWCNT solvation shell, the second SWCNT solvation shell and beyond the second SWCNT solvation shell (>12.2 Å and >11.9 Å from the SWNCT wall for NMP and DMAc, respectively). **b**, Snapshot of EPSR ensemble local to the SWCNT showing sodium atoms with highlighted solvation shell radii. **c**, SDFs of sodium around solvent molecules, at 50% threshold.

With this in mind, we examine the distribution of sodium cations around the negatively charged SWCNT. Although only present in relatively dilute concentrations (0.175 mol dm^−3^), the sodium cations strongly interact with solvent molecules that themselves are more substantially weighted in the diffraction-derived model. We therefore place a greater weight in our analysis to the sodium locations with respect to the solvent molecules. The sodium cRDF (Fig. 4a) indicates that the sodium ions are arranged in spatially distinct relative positions around the nanotube: ~17% are adsorbed in a narrow band ~4 Å from the SWCNT surface with the rest distributed at further distances. Classical Monte Carlo simulations of sodium ions around charged nanotubes in a simple dielectric medium (that is, not accounting for local solvent structure) predict notably higher degrees of sodium adsorption, regardless of the dielectric value (Supplementary Discussion 3). Although comprehensively understanding long-range sodium correlations is beyond the scope of this study, locally we observe that ions are less commonly located in the centre of the nanotube solvation shells, where solvent density is highest (Figs. 1e and 4b). We suppose that ions are excluded from these regions because they would disrupt the greater intersolvent structure inherent to higher-density regions.

Analysis of amide–sodium SDFs (Fig. 4c) reveals the nature of the solvent–cation interactions for both condensed and non-condensed sodium ions in both solvents. In the bulk solvent, the sodium ions are fully solvated by the amides, which direct their oxygen atoms towards the positive ion, while surface-adsorbed sodium ions show less-ordered correlations to the solvent, with local amide molecules preferentially coordinating to the SWCNT.

## Conclusions

The atomistically resolved measurements presented here uncover a number of important insights. The observed solvent ordering is complex, involving four solvation shells up to at least 20 Å from the surface (Fig. 1c), and orientational ordering which extends beyond these density fluctuations out to 40 Å from the SWCNT (Fig. 2c). The ordering is dictated by competing effects at different length scales with the solvent orientation influenced by the SWCNT at distances even beyond the density fluctuations of the solvation shells. Despite their substantial rearrangement in accommodating the SWCNTs, the solvent ordering is realised while largely maintaining the average intersolvent structure found in the pure solvent, thus mitigating the overall entropic cost associated with the dissolution of the SWCNTs^26,32^. The intricately ordered solvent substantially differs from the picture in classical models designed for understanding colloidal dispersions or ion ordering at surfaces in electrochemical devices^9^. The results highlight the critical importance of including multibody effects and the 3D nature of solvent molecules for describing these systems^7,11,33^.

The results also reveal an experimental picture of the electrical double-layer model found at charged surfaces. An adsorbed Stern-like layer of desolvated counterions at the charged SWCNT surface is driven by the strong Coulombic attraction between negative surface and positive counterion, beyond which a diffuse layer of solvated ions is observed, leaving a net negative charge. Our findings therefore have important implications for understanding electrochemical devices based on SWNTs and other nanostructured *sp*^2^ carbon surfaces including supercapacitors^35^, capacitive deionization membranes^36^, batteries and electrolytically gated transistors^37^. For supercapacitors, enhanced capacitance upon reduction of pore size to below the solvation shell of the ions has previously been explained by their desolvation upon adsorption^14^. Our work, however, shows that at the (convex) charged nanotube surface, solvent–interface ordering occurs in preference over sodium coordination, suggesting that these ions are already ‘desolvated’ within the surface-ordered solvent region due to competing effects, independent of confinement. However, it should be noted that in our model system there are no ions with the same charge as the surface, which may also have an important effect^15,16^. Overall, our results reveal the critical importance of fully describing the solvent for understanding such systems, and the judicious choice of solvent alongside pore and ions size for improving performance, and highlight wide-angle neutron scattering as an important tool for investigating these systems.

## Methods

### Materials

HiPco SWCNTs were purchased from Nanointegris (Super Purified, containing <5 wt% residual iron catalyst) and outgassed at ∼10^−7^ mbar at 300 °C overnight before use. Sodium metal (99.95%, ingot), D_9_-DMAc/D_9_-NMP (≥99.5 at.% D) and anhydrous DMAc (99.8%) were obtained from Sigma Aldrich. Anhydrous ammonia (99.98%) was purchased from BOC Industrial Gases and was purified before use by condensing over sodium metal.

### Nanotubide salt synthesis

In a high-purity-argon-filled glovebox (O_2_, H_2_O <0.1 ppm), SWCNTs (144 mg, 12 mmol assuming all carbon) and sodium metal (23 mg, 1 mmol) were placed in a glass reaction tube fitted with a metal Swagelok valve. The tube was sealed, removed from the glovebox and cooled to 223 K before exposing to ammonia gas until sufficient NH_3_ had condensed to cover the SWCNT powder. The liquid quickly turned deep blue due to the presence of solvated electrons, before turning black over 2 h (from dissolution of SWCNTs), resulting in a sodium nanotubide–ammonia solution^38^. The ammonia was then slowly removed by cryopumping, before drying under vacuum (<5 mbar) and the resultant sodium nanotubide salt transferred to the glovebox without exposure to air. Elemental concentrations were determined by CHN analysis using an Exeter Analytical CE440 Elemental Analyser. Combustion of the sample, separation of the combustion gases and measurement by thermal conductivity were all carried out in dynamic mode. Duplicate measurement runs agreed to within 0.5%. The final stoichiometry was C_12_(NH_3_)_1.2_Na, constituting the carbon-only SWCNT anions, remnant ammonia and sodium counterions, respectively.

### Neutron scattering experiments

A fuller description of neutron scattering theory is provided in Supplementary Discussion 1. Neutron diffraction experiments were performed using the Near and InterMediate Range Order Diffractometer (NIMROD) instrument at the ISIS spallation neutron source (Rutherford Appleton Laboratory, UK)^24,39^. This purpose-built diffractometer was optimized for structural studies of hydrogen-containing disordered materials. Data were obtained for nanotubide salt dissolved in NMP and DMAc, respectively (25 mg (NaC_12_(NH_3_)_1.2_) ml^−1^) to form high-viscosity fluids using three isotopomeric systems: non-deuterated DMAc/NMP, fully deuterated DMAc/NMP and a 1:1 molar mixture of the two monoisotopic solvents. Samples were loaded into flat-plate null-coherent scattering Ti_0.68_Zr_0.32_ alloy cells of internal dimensions 1 mm × 35 mm × 35 mm (that is, 1.225 ml). In an argon-filled glovebox, ∼27.5 mg of sodium nanotubide powder was added to the cell and weighed before solvent was added. For hydrogenated solvents 0.04 ml mg_(NaSWCNT)_^−1^ was added, using solvent weight to measure the correct stoichiometry. For 50:50 H/D solvents, equimolar solvents were premixed. Cells were sealed with custom PFTE seals before removal from the glovebox and leak checking under vacuum. Sealed cans were then sonicated in a bath sonicator for 1 h before measurements. Unsealing of the cans after measurements revealed homogeneous black gel, and measurements did not demonstrate Bragg peaks associated with SWCNT bundles, indicating the success of the dissolution process. Neutron diffraction data were collected over the range 0.02 Å^−1^ < **Q** < 50 Å^−1^, where **Q** is the scattering vector. Once loaded onto the sample changer, the temperature was maintained at 21 °C. Scattering from the empty instrument and with empty sample cells was measured for data-correction purposes. The sample data were normalized, calibrated and put on an absolute scale by comparison with the scattering from a 3 mm null-coherent-scattering vanadium–niobium plate. Particular attention was paid to correction of inelasticity effects, especially for the samples containing hydrogen. The self-scattering background and inelasticity effects were removed from the total differential scattering cross section using an iterative method developed by Soper^23^.

### EPSR modelling

A fuller description of EPSR is provided in Supplementary Discussion 1. An equilibrated Monte Carlo ensemble of the system was used as a starting point, seeded by pairwise (Lennard–Jones and Coulomb) potentials (Supplementary Table 1). Two intertorsional angles (dihedrals) were defined within the DMAc/NMP molecule to describe its skeletal planarity, as suggested by previous findings in the literature^26^. The hydrogen atoms of methyl groups attached to the nitrogen atom were allowed to freely rotate around the C–N axis. The standard minimum image convention was applied to the ensemble with appropriate periodic boundary conditions. The simulation boxes for the two systems comprised an orthorhombic box of sides 104.4 Å × 104.4 Å × 48.57 Å for the nanotubide dissolved in NMP and 105.07 Å × 105.07 Å × 48.57 Å in DMAc. The simulation boxes contained 42 sodium atoms (+1*e* charge each), 51 ammonia molecules, 3,297 NMP molecules/3,424 DMAc molecules and 1 SWCNT fixed along the 48.57 Å axis (508 atoms, −42*e* charge, evenly distributed, providing a negative surface charge of ~0.032 *e* Å^−2^) to match that of C_12_(NH_3_)_1.2_Na at 25 mg ml^−1^. The ammonia component was used from previous CHN analysis of nanotubide salts showing a 1:1.2 ratio of Na:NH_3_ remaining from lithium electride reduction of nanotubes. A (7,6) chirality nanotube was used in the model, which is the dominant chiral species in the HiPco sample used. It should be noted that while (7,6) is an intrinsically semiconducting chirality, in the reduced state both (7,6) SWCNTs and heterogeneous HiPco samples overall are metallic^40^. Simulations were run at 298 K using standard Metropolis–Hastings Monte Carlo steps to bring the system to equilibrium. Once equilibrated, the EPSR procedure was invoked whereby an iterative refinement of site–site interatomic potentials drives the simulated structure toward agreement with the experimental neutron diffraction data. Modelled total structure factors were derived from calculated composite RDFs of the model cell using a Fourier transform with a maximum **Q** limit of 30 Å^−1^. Once a good agreement was achieved, the simulation proceeded without further perturbation of the potentials and ensemble-average structural information was then accumulated.

cRDFs are calculated radially normal to the central SWCNT axis vector, with radii subsequently offset by 4.47 Å—the atom-centre to nanotube-centre radius of a (7,6) SWCNT—to provide distances to the SWCNT carbons. These offset radii are used for binning orientational data as a function of distance from the SWCNT. Note that although cRDFs are conceptually similar to RDFs, direct comparisons should be treated with caution: RDFs sample a 3D sphere from a defined point and cRDFs sample a two-dimensional cylinder normal to the central vector and therefore require different methods for normalization. CoM were calculated using dlputils, averaging the position of non-H atoms. Spatial density functions are plotted about a calculated average solvent molecule, sampled with 0.1 Å increments measured spherically from the CoM from −10 to +10 Å, presenting 15% of most likely positions for solvent–solvent SDFs, and 50% of most likely positions for solvent–sodium SDFs. Angular RDFs were measured for the angular difference between the carbon nanotube central axis and a solvent axis; either a dipole-parallel *x* axis along the C=O bond or an out-of-plane *z* axis (accurately defined as the cross-product of the *x* axis and *y* axis defined by the amide C–N vector). For each molecule, the minimum distance between the CoM and the nanotube vector was calculated, and the dot product between the SWCNT-perpendicular vector and each axis of the molecule was summed into distance bins of 0.1 Å and angle bins of 5° to give angle versus distance data. Data were normalized within each summed distance bin to give distribution per solvent molecule per angle bin (that is, with equal population of all angles giving a population of 1 in all angle bins).

## Online content

Any methods, additional references, Nature Portfolio reporting summaries, source data, extended data, supplementary information, acknowledgements, peer review information; details of author contributions and competing interests; and statements of data and code availability are available at 10.1038/s41565-025-01865-9.

## Supplementary information

Supplementary Information Supplementary Figs. 1–12, Discussions 1–4 and Tables 1–10.

## Figures and Tables

**Extended Data Fig. 6:**
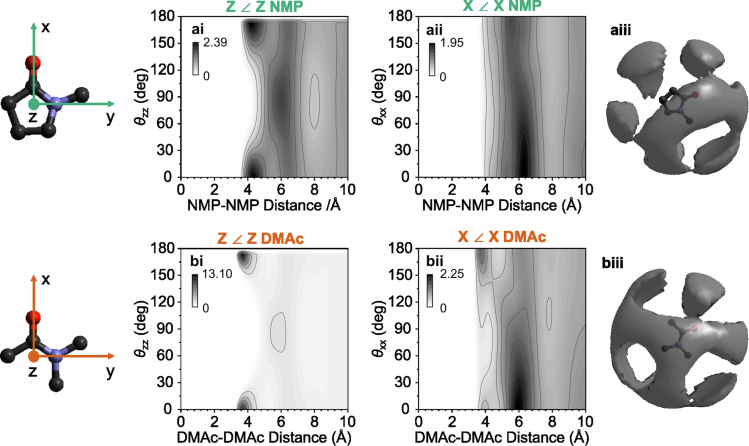
Solvent–solvent relative orientation for all solvent molecules in analysis cell. aRDFs of NMP (**a**) and DMAc (**b**) with defined axes (top) of x along the C = O axis that approximates the direction of the dipole and z normal to the molecular plane. Inter-z (i) and inter-x (ii) angles, binned in 10° increments. The *g(r,θ)* intensity is given by greyscale inset in each graph. (iii) Amide–amide SDFs at of 15% of most likely positions.

**Extended Data Fig. 7:**
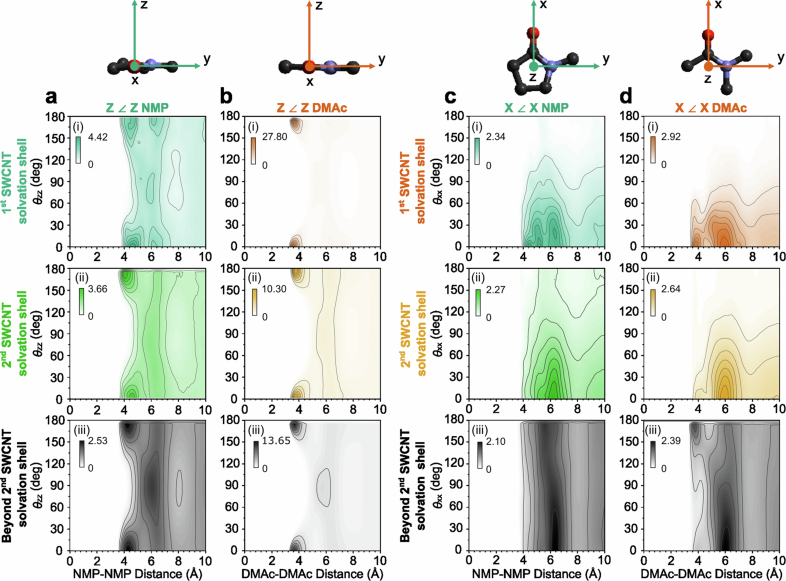
Solvent–solvent relative orientation in the 1st solvation shell, 2nd shell, and beyond the SWCNT solvation shells, scaled to cover the full g(r) range of each individual aRDF. Angular RDFs of NMP (**a, c**) and DMAc (**b, d**) with defined axes (top) of x along the C = O axis that approximates the direction of the dipole and z normal to the molecular plane. Inter-z (*θ*_*zz*_, **a, b**) and inter-x (*θ*_*xx*_, **c, d**) angles, binned in 10° increments. Data sampled from solvents with CoM found within (i) 1st SWCNT solvation shell (NMP-SWCNT < 5.9 Å; DMAc-SWCNT < 6.1 Å), (ii) 2nd SWCNT solvation shell (NMP-SWCNT 5.9–12.1 Å; DMAc-SWCNT 6.1–11.9 Å), and (iii) beyond the second SWCNT solvation shell (>12.2 Å and > 11.9 Å from the SWNCT wall for NMP/DMAc, respectively). The *g(r,θ)* intensity is given by colour scale inset in each graph.

## Data Availability

Raw diffraction data are publicly available at 10.5286/ISIS.E.RB1910503 (ref. ^39^). The raw data for the pure-solvent data of NMP and DMAc (experiment and analysis published previously^26,32^) are publicly available at 10.5286/ISIS.E.24079795 and 10.5286/ISIS.E.RB1700030, respectively.
