# Supplementary material for: Intermediate-range solvent templating and counterion behaviour at charged carbon nanotube surfaces
Source: Nat Nanotechnol. 2025 Feb 21;20(5):639–45. doi: 10.1038/s41565-025-01865-9 (PMC12095044; doi:10.1038/s41565-025-01865-9)
Supplement: Supplementary file 1 — Supplementary Figs. 1–12, Discussions 1–4 and Tables 1–10. [file 41565_2025_1865_MOESM1_ESM.pdf]

nature nanotechnology

Supplementary information https://doi.org/10.1038/s41565-025-01865-9

Intermediate-range solvent templating and counterion behaviour at charged carbon nanotube surfaces

In the format provided by the authors and unedited

Table of Contents

Supplementary Discussion 1. Neutron Diffraction Theory & EPSR Molecular Modelling	2
Supplementary Discussion 2. Scattering of Residual Ammonia and Iron	20
Supplementary Discussion 3. Classical Monte Carlo simulation of Na-SWCNT salt in a Dielectric Medium	25
Supplementary Discussion 4. Comparison of Model to X-Ray Scattering	30
Supplementary References	34

Supplementary Discussion 1. Neutron Diffraction Theory & EPSR Molecular Modelling

Neutron scattering experiments measure the differential scattering cross-section, on an absolute scale through calibration with scattering from a vanadium-niobium plate[S1,S2] after accounting for baseline scatter from background instrumental and sample cell effects. Following these corrections, the total structure factor, $F(Q)$, which provides a direct measurement of the structure in reciprocal space, can be extracted. Q is the magnitude of the momentum change vector of the scattered neutrons and is given by:

$$Q = \frac{4\pi}{\lambda}\, sin\theta \qquad \text{(Eq. 1)}$$

where λ is the wavelength of neutrons incident onto the sample and 2θ is the scattering angle.

F(Q) can be written as a sum of the Faber-Ziman partial structure factors, $S_{\alpha\beta}(Q)$:

$$F(Q) = \sum_{\alpha \le \beta} \left(2 - \delta_{\alpha\beta}\right) c_\alpha c_\beta b_\alpha b_\beta \left(S_{\alpha\beta}(Q) - 1\right) \qquad \text{(Eq. 2)}$$

weighted by the respective concentrations, c_α and c_β, and scattering lengths, b_α and b_β, of each atom type.[S3] The Kronecker delta function, $\delta_{\alpha\beta}$, is used to avoid double counting. The $S_{\alpha\beta}(Q)$ terms contain information about correlations between the atomic species in Q-space, and can be related to the radial distribution functions, $g_{\alpha\beta}(r)$, via a Fourier transformation:

$$S_{\alpha\beta}(Q) = 1 + 4\pi\rho \int_0^\infty r^2\, [g_{\alpha\beta}(r) - 1]\, \frac{sin\,(Qr)}{Qr} dr \qquad \text{(Eq. 3)}$$

where ρ is the atomic density of the sample.

The key aim of most structural studies of liquids is to extract $g_{\alpha\beta}(r)$, the function representing real space correlations between atom pairs as a function of the separation, r, between them. The cumulative coordination number of species β from species α at a distance r is given as $N_{\alpha\beta}(r)$ and can be found using:

$$N_{\alpha\beta}(r) = \int_0^r g_{\alpha\beta}(r')\, \rho_\beta \cdot 4\pi r'^2 dr' \qquad \text{(Eq. 4)}$$

Measurement of several isotopomeric samples varies the contrast of the total structure factor, allowing for multiple diffraction patterns of a specific system to be measured.

In order to interpret the data and maximize the extracted information from the neutron diffraction, empirical potential structure refinement (ESPR) was carried out[S4,S5]. This procedure produces a three-dimensional ensemble model of the system consistent with the measured scattering data[S1,S6-9]. A combination of Lennard-Jones (LJ) and Coulomb potentials were used to build the following reference potential, $U_{\alpha\beta}(r)$, between atom pairs, α and β, in the molecule, represented by:

$$U_{\alpha\beta}(r) = 4\varepsilon_{\alpha\beta}\left[\left(\frac{\sigma_{\alpha\beta}}{r}\right)^{12} - \left(\frac{\sigma_{\alpha\beta}}{r}\right)^{6}\right] + \frac{1}{4\pi\varepsilon_0}\frac{q_\alpha q_\beta}{r} \qquad \text{(Eq. 5)}$$

where $\varepsilon_{\alpha\beta}$ and $\sigma_{\alpha\beta}$ are computed using the classical Lorentz-Berthelot mixing rules for the cross terms,[S10] and ε_0 is the permittivity of free space. Initial seed potentials are provided in Supplementary Table 1.

Supplementary Table 1. Reference Seed Potentials for all of the labelled atoms in the EPSR model of $Na(NH_3)C_{12}$ in amidic solvent, showing atom labels, Lennard-Jones parameters and charge. [S11,S12]

Species	Atom Labels	Atom	ϵ / kJ mol^{-1}	σ / Å	q / e
(7,6) SWCNT	C	C	0.293	3.55	-0.0827
NMP	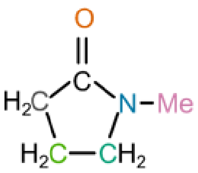	O	0.87864	2.96	-0.50
		C	0.43932	3.75	+0.50
		N	0.71128	3.25	-0.20
		C_{Me}	0.27614	3.50	-0.13
		$(N)\underline{C}H_2(CH_2)$	0.27614	3.50	-0.07
		$(CH_2)\underline{C}H_2(CH_2)$	0.27614	3.50	-0.07
		$(CO)\underline{C}H_2(CH_2)$	0.27614	3.50	-0.07
		H_{Me}	0.12552	2.50	+0.06
		$(N)C\underline{H_2}(CH_2)$	0.12552	2.50	+0.06
		$(CH_2)C\underline{H_2}(CH_2)$	0.12552	2.50	+0.06
		$(CO)C\underline{H_2}(CH_2)$	0.12552	2.50	+0.06
DMAc	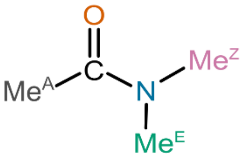	O	0.87864	2.96	-0.50
		C	0.43932	3.75	+0.50
		N	0.71128	3.25	-0.14
		C^Z	0.27614	3.50	-0.11
		C^E	0.27614	3.50	-0.11
		C^A	0.25104	3.50	-0.18
		H^Z	0.12552	2.50	+0.06
		H^E	0.12552	2.50	+0.06
		H^A	0.12552	2.50	+0.06
Sodium	Na^+	Na	6.72	0.189	+1.0
Ammonia	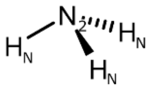	N_2	0.71128	3.42	-1.02
		H_N	0.0	0.0	+0.34

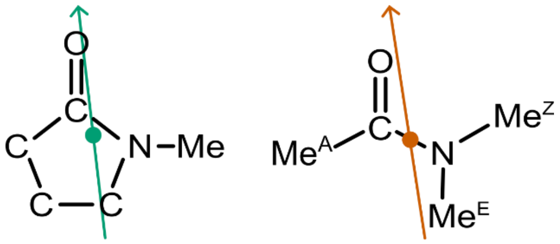

Supplementary Figure 1. Dipole moment directions for NMP and DMAc molecules. A circular dot highlights the center-of-mass for the two molecules. N.B., Here, dipoles are defined as pointing from positive to negative regions.

The EPSR process begins with a standard Monte Carlo simulation, with traditional implementation of periodic boundary condition and the minimum image convention. A smooth truncation is applied to the Lennard-Jones potential energy functions, as detailed by Soper,[S4] using a function of the form:

$$T(r) = \begin{cases} 1 & r \leq r_1 \\ 0.5\left[1 + cos\pi\left(\frac{r - r_1}{r_2 - r_1}\right)\right] & r_1 < r < r_2 \\ 0 & r \geq r_2 \end{cases} \quad \text{(Eq. 6)}$$

where r_1 = 20 Å and r_2 = 23 Å. The Coulomb potentials are also truncated using the Local Molecular Field method:

$$T_c(r) = erfc\left(\frac{r}{\sigma_c}\right) \quad \text{(Eq. 7)}$$

derived by Chen and Weeks where σ_s is the largest of 3.5 Å and (1/5)·r_{maxpt}. [S13]

To better reproduce the experimental data, and to optimize computational resources, multiple initial configurations were run using different distributions of sodium ions about the central nanotube. Excluding systems with (near) full condensation of sodium – which are physically unrepresentative given the known dissolution of the nanotubide salt – we compared the quality of the fit with relatively little difference seen (Supplementary Figure 2b). We therefore selected the system with 14% of sodium ions initially in close proximity (≤ 9 Å of the SWCNT central axis) to the CNT, which gave marginally the best agreement with experimental data, as most apparent from the R-factors (Supplementary Figure 2a). We also note that the final configuration does change in total number of ions condensed (17%). However, given the low weighting of the CNT-Na and Na-Na partial structure factors and similarities in fitting quality between initial configurations, we do not consider the fraction of sodium condensed in our analysis to be quantitative. The structural information of the system was extracted from a trajectory file where over 50,000 configurations were collected via the *dlputils* routines and visualized using the *Aten* software. [S14,S15]

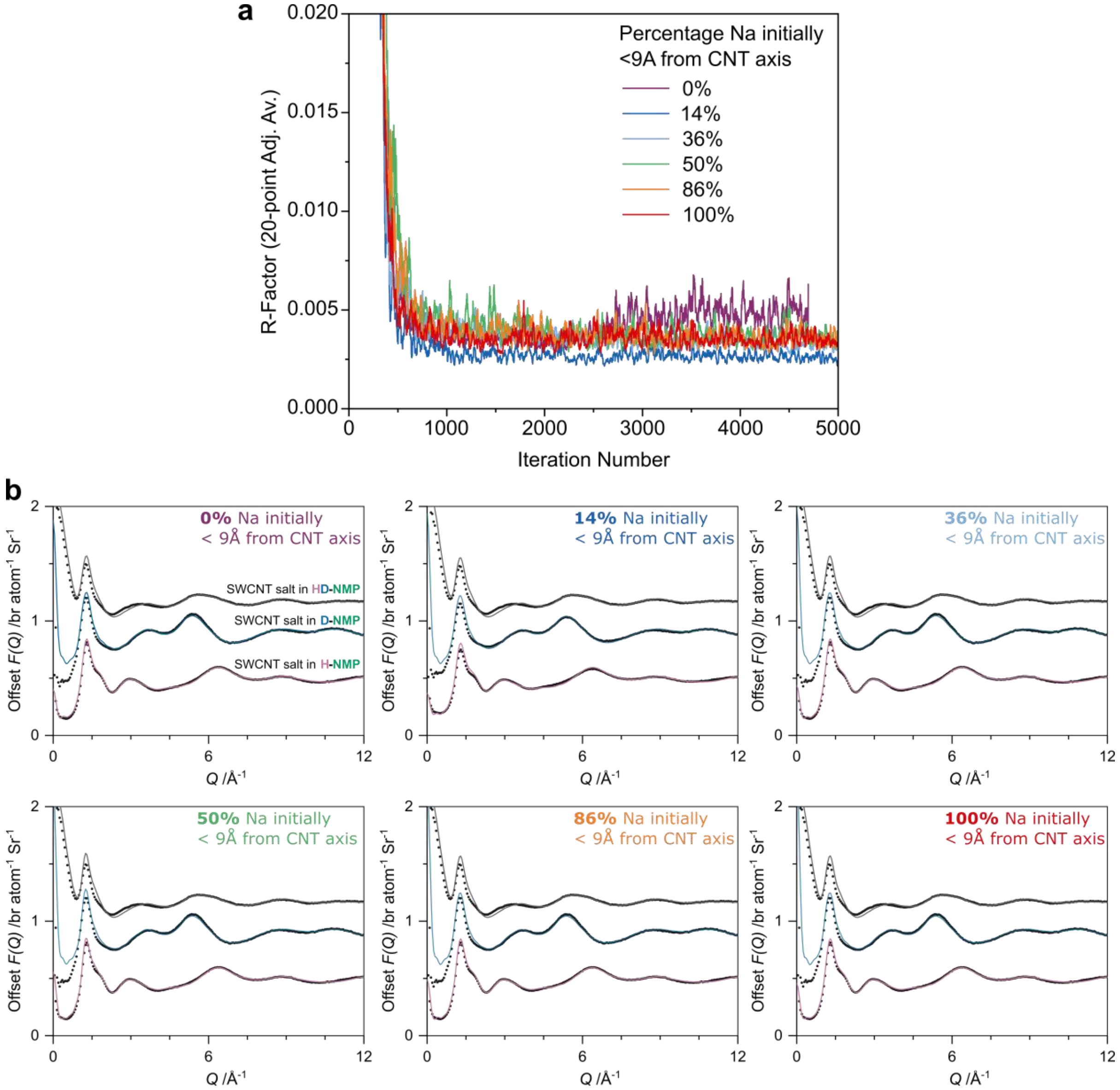

Supplementary Figure 2. (a) R-factor quality-of-fit values for EPSR fitting iterations for model systems with initial geometry consisting of a predefined fraction of sodium atoms initially placed within 9 Å of the SWCNT central axis (4.53 Å of the SWCNT wall). Smoothing with 20-point adjacent averaging used for visual clarity. (b) Total structure factors from neutron scattering (black circles) and model system (solid line) with initial geometry consisting of a predefined fraction of sodium atoms initially placed within 9 Å of the SWCNT central axis (4.53 Å of the SWCNT wall). From top to bottom, HD-NMP, D-NMP, and H-NMP, plotted with y-offsets of 1.17, 0.9, and 0.5, respectively for clarity.

The EPSR modelled total structure factors for the nanotubide solutions capture well the key features of the experimental data across the full Q-range accessible via

NIMROD, as highlighted by the low R-factors (~2.5×10^{-3}, Supplementary Figure 2a). Discussing the key regions in turn:

- The intramolecular region (Extended Data Figure 1-2, $Q \gtrsim 3$ Å^{-1}) shows excellent peak centre and amplitude agreement for all solvent isotopic contrasts with only very minor intensity discrepancies, attributed to assumptions in the intramolecular modelling of bond vibration and torsion.[S4,S5] The clear agreement here can also be seen in the fits to the real space G(r).

- The intermolecular nearest-neighbour region (Extended Data Figure 1-2, $1 \lesssim Q \lesssim 3$ Å^{-1}, $r \gtrsim 2$ Å) show good agreement. The fit captures the key peak positions very well, with some slight deviation in peak intensity, particularly when compared to the excellent fit obtained for the neat solvents (Supplementary Fig 3-4, with comparison of residuals in Supplementary Fig 5a). This slight weakening of the fit in the more complex nanotubide system is to be expected due to the necessary assumptions and simplifications of the EPSR refinement model when compared to the experimental solution (for example SWCNT chirality and ends without residual iron).
- Intermolecular Intermediate-range region (beyond the nearest-neighbours, Extended Data Figure 1-2, $Q \lesssim 1$ Å^{-1}, $r \gtrsim 6$ Å): The minor differences between EPSR-fit and measurement as noted above continue in this region. However, notably, in this region where intermediate range interactions are represented, the differences between EPSR-modelled and experimental SWCNT solution data are smaller than those between measurements of the SWCNT solution and pure solvent for hydrogenated and deuterated samples, and highly comparable for 50:50 HD samples (Supplementary Figure 5b). These data indicate that the EPSR procedure is successfully constraining the simulation by adequately

refining the pair-potentials to capture the intermediate-range solvent structure while simultaneously accounting for molecular- and atomic-packing regime (higher-Q) interactions.

Scattering contributions of each molecule/atom type for each fully hydrogenated/deuterated system provided in Supplementary Tables 4-7. For analyzing the molecular arrangements within the system, a vector describing the direction along which the nanotube lays was used, along with a point (on which this vector lays) to define the center of the nanotube. A central vector (0, 0, 1) was defined from the geometric center of the nanotube at (0, 0, 0) to a point along the nanotube central axis. The NMP/DMAc molecules x-axis was defined along the C=O axis which is also the closest vector to the molecular dipole (Supplementary Figure 1). The y-axis set along a N-C(methyl) bond, with the z-axis (the cross product of x/y-axes) lying out of plane of the amidic C=O-N plane. For each molecule, the minimum distance (that is the perpendicular distance) between the center-of-mass and the defining vector was calculated, and the dot product between the SWCNT-perpendicular vector and each axis of the molecule was summed into distance bins of 0.1 Å and angle bins of 5° to give angle versus distance data. The distance bins were summed between the solvation shell minima (taken from solvent cylindrical distribution functions, cRDFs) and as 5 Å tranches thereafter, before normalized to give distribution per solvent molecule per angle bin (i.e., with equal population of all angles giving a population of 1 in all angle bins). SDFs were plotted about a calculated average solvent molecule, sampled with 0.1 Å increments from -10 to +10 Å, presenting 15% of most likely positions for solvent-solvent SDFs, and most likely 50% positions for solvent-sodium SDFs.

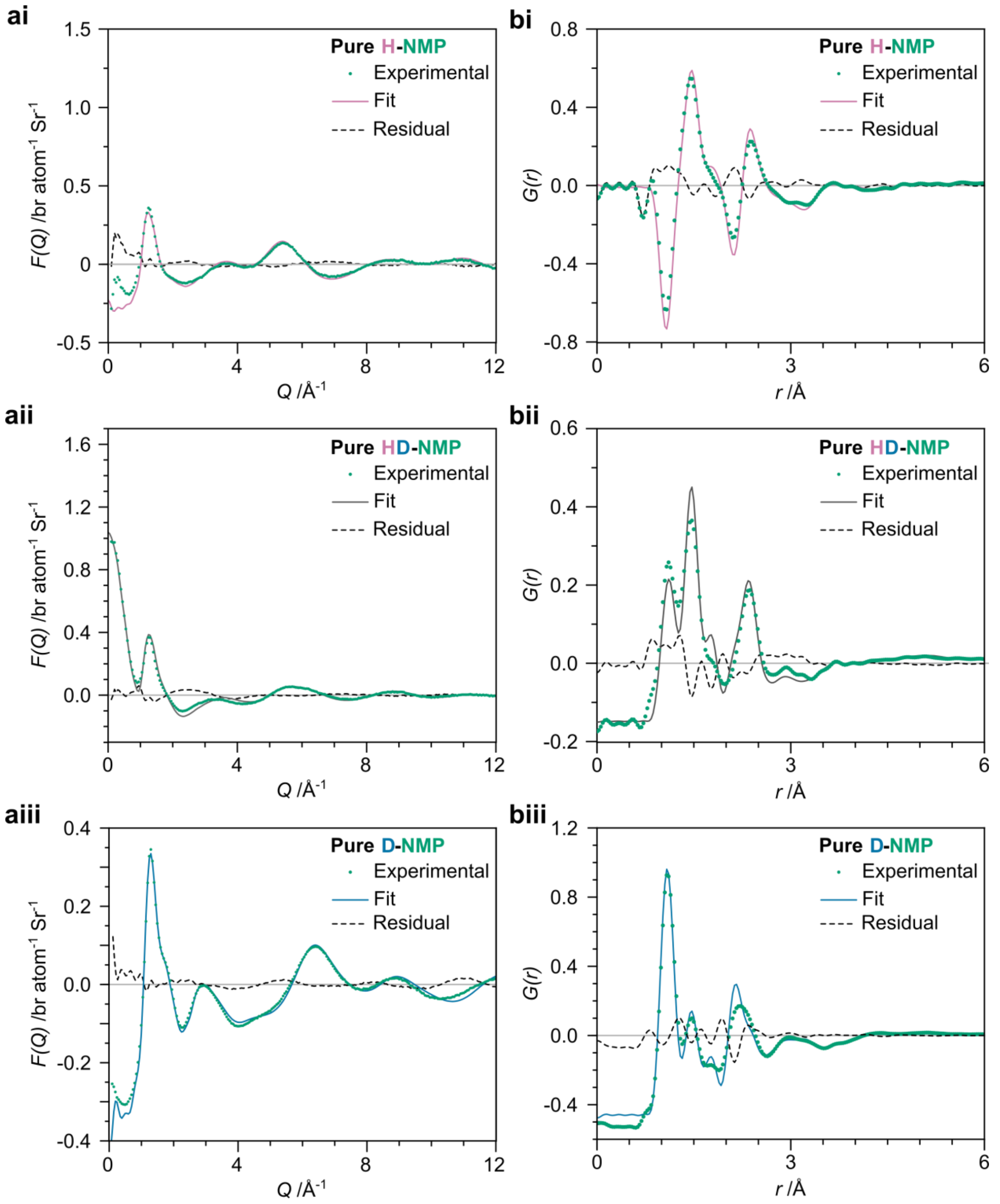

Supplementary Figure 3. (a) Total structure factors and (b) Composite radial distribution functions of pure NMP (i) H-NMP, C5H9NO (ii) 50:50 molar mixture of H-NMP and D-NMP, (iii) D-NMP, C5D9NO. Green circles – Experimentally acquired data; Solid line – Data from EPSR-refined simulation using the same seed potentials as the CNT-NMP analysis; Black dashed line – Residual (Fit-Experimental). Guide to the eye at y = 0 provided as pale grey line for comparison to residual. Neutron data available from ISIS repository (doi:10.5286/ISIS.E.RB1010464).

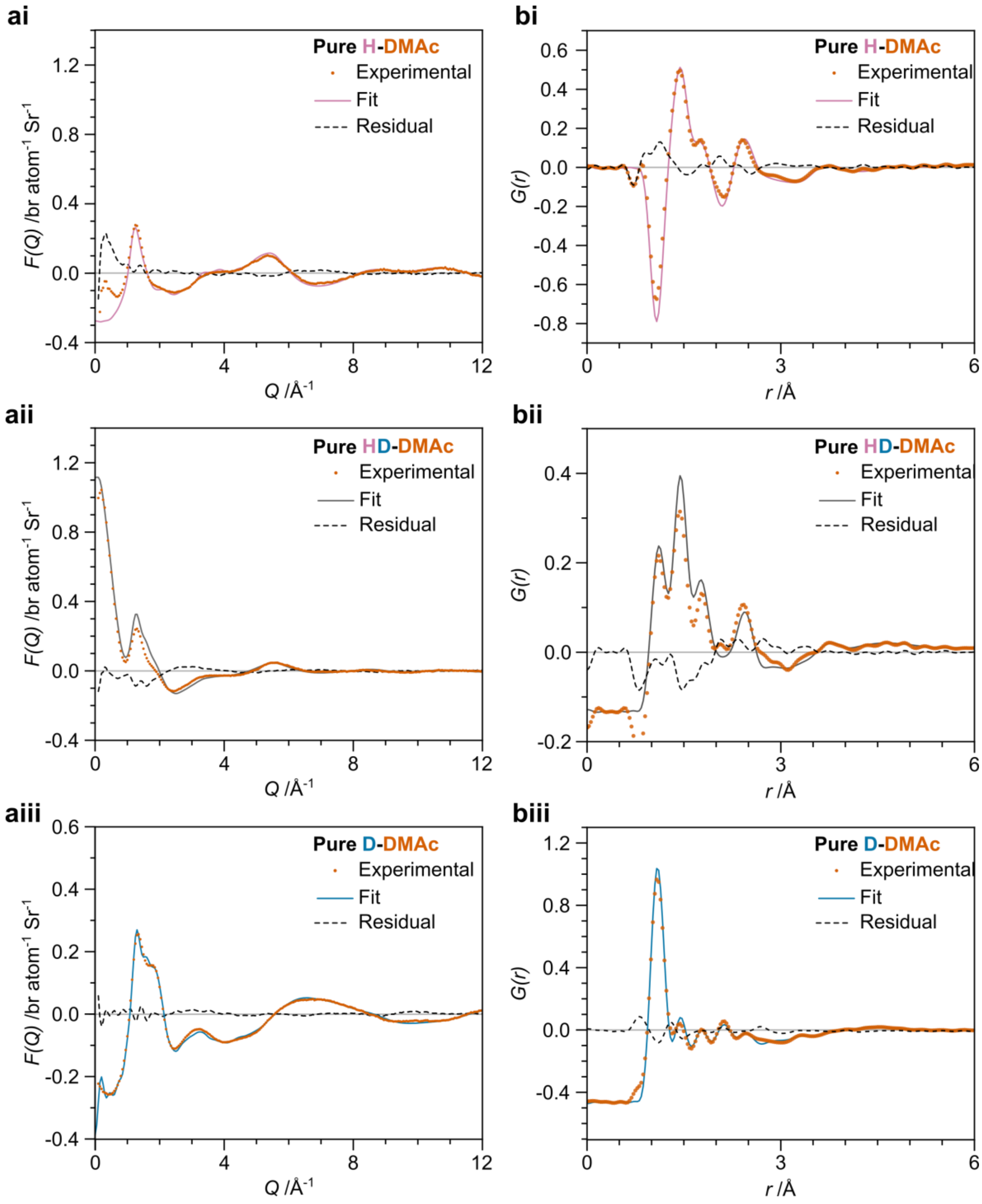

Supplementary Figure 4. (a) Total structure factors and (b) Composite radial distribution functions of pure DMAc (i) H-DMAc, C_4H_9NO (ii) 50:50 molar mixture of H-DMAc and D-DMAc, (iii) D-DMAc, C_4D_9NO. Vermillion circles – Experimentally acquired data; Solid line – Data from EPSR-refined simulation using the same seed potentials as the CNT-DMAc analysis; Black dashed line – Residual (Fit-Experimental). Guide to the eye at y = 0 provided as pale grey line for comparison to residual. Neutron data available from ISIS repository (doi:10.5286/ISIS.E.RB1700030)

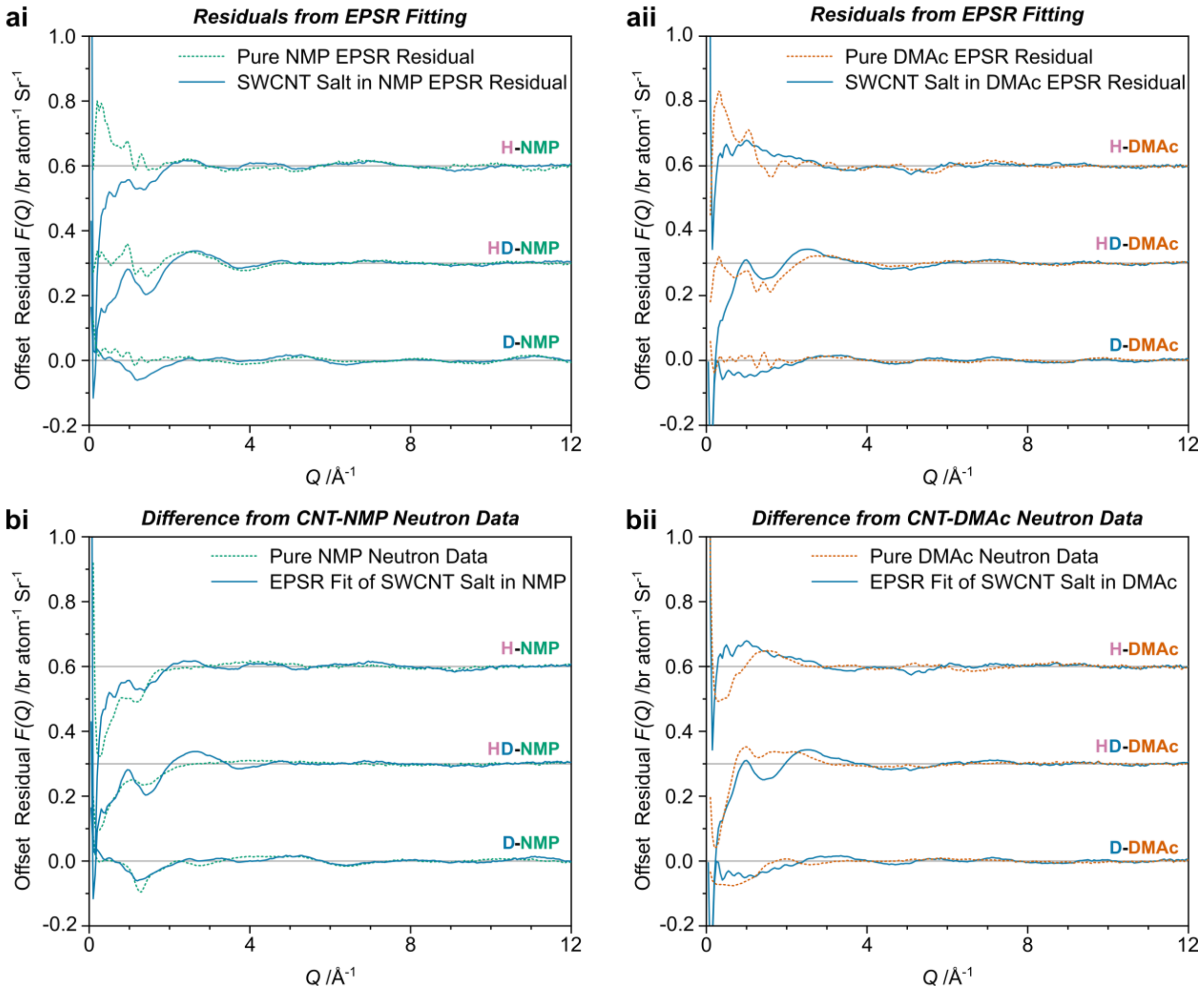

Supplementary Figure 5. Comparison of quality of fit. (a) Residual of the total structure factor from EPSR refined modelling of (green/orange dotted) pure solvent, n.b., structures not presented in this work, and (blue solid) CNT-solvent systems, using the same input solvent geometry and seed forcefield parameters. Isotopic contrasts offset by D = 0, HD = 0.3 and H = 0.6. Guide to the eye at d(F(Q)) = 0 for each offset contrast provided as pale grey line for comparison. (b) Difference from CNT-solvent total structure factor measured via neutron scattering, taken by subtracting: (green/orange dotted) pure solvent total structure factor measured via neutron scattering, and (blue solid) EPSR fit of CNT-solvent system. Isotopic contrasts offset by D = 0, HD = 0.3 and H = 0.6. Guide to the eye at d(F(Q)) = 0 for each offset contrast provided as pale grey line for comparison.

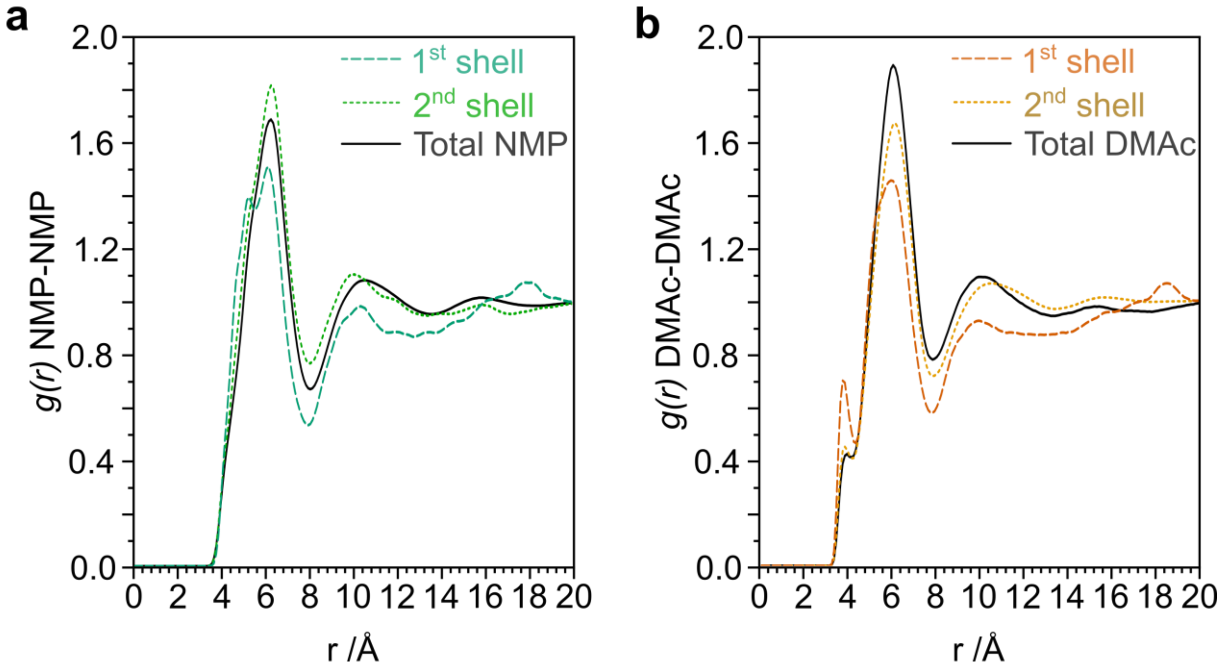

Supplementary Figure 6. (b) amide-amide center-of-mass RDFs within the first solvation shell (turquoise/orange dash), 2nd – 3rd solvation shell (green/orange dotted) and all solvent (black solid) for (a) NMP, and (b) DMAc.

Supplementary Table 2. Calculated NMP densities per solvation shells around SWCNT. (a) Box height is 47.8 Å. (b) Using total box volume (104.40 Å × 104.40 Å × 48.57 Å) and number of NMP minus volume/NMP molecules of 1st – 4th shells. N.B., shell limits are taken here from the SWCNT central axis, without the 4.47 Å offset used to provide distances to the SWCNT wall.

Shell	Shell Limits (Å)		Shell Volume[(a)] ($Å^3$)	# NMP	% NMP	NMP per $Å^3$	% Density Change vs Bulk
	Inner	Outer					
1st	7.45	10.35	7876.5	91.5	2.8	0.01162	+85.6
2nd	10.35	16.55	25448	143.7	4.4	0.00565	-9.7
3rd	16.55	21.55	29068	180.9	5.5	0.00622	-0.5
4th	21.55	26.35	35083	225.2	6.8	0.00642	+2.6
Non-Solvation Shell[(b)]	n/a	n/a	431906	2655.7	80.5	0.00615	-1.7
Bulk NMP						0.00626	0.0

Supplementary Table 3. Calculated DMAc densities per solvation shells around SWCNT. (a) Box height is 47.8 Å. (b) Using total box volume (105.07 Å × 105.07 Å × 48.57 Å) and number of DMAc minus volume/DMAc molecules of 1st – 4th shells. N.B., shell limits are taken here from the SWCNT central axis, without the 4.47 Å offset used to provide distances to the SWCNT wall.

Shell	Shell Limits (Å)		Shell Volume[(a)] ($Å^3$)	# DMAc	% DMAc	DMAc per $Å^3$	% Density Change vs Bulk
	Inner	Outer					
1st	7.45	10.55	8514.4	100.0	2.9	0.01175	+80.8
2nd	10.55	16.35	23807	143.0	4.2	0.00601	-7.5
3rd	16.35	21.45	29416	183.2	5.4	0.00623	-4.1
4th	21.45	26.35	35739	232.2	6.8	0.00650	0.0
Non-Solvation Shell[(b)]	n/a	n/a	536198	2765.5	80.8	0.00630	-2.0
Bulk DMAc						0.00650	0.0

Supplementary Table 4. Fractional atom-pair neutron scattering contribution (Eq. 8) for 25 mg mL^{-1} SWCNTs in D-NMP. Contributions summed for each atom/species type and presented as percentage contributions. Red-yellow-blue gradient colour scale used within fractional atom-pair scattering contribution table, atom-type contribution column, and species contribution column, respectively, to provide visual aid for identifying relative scattering contributions. Neutron scattering cross sections taken from Sears[S3].

			Species	Na	Ammonia		CNT		D-NMP				Percentage Scattering Contribution	
					N	H	C	Fe	C	O	N	D		
			Atom Fraction	0.000018	0.000018	0.000163	0.009008	0.0001	0.309568	0.01913	0.01913	0.55722		
Species		Atom Fraction	Coherent Scattering Length (fm)	3.63	9.36	-3.739	6.646	9.45	6.646	5.803	9.36	6.671		
Na		0.000018	3.63	1.14E-10	2.93E-10	1.06E-09	1.04E-07	1.65E-09	3.58E-06	1.93E-07	3.12E-07	6.47E-06	0.001%	0.001%
NH_3	N	0.000018	9.36	2.93E-10	7.56E-10	2.74E-09	2.69E-07	4.24E-09	9.24E-06	4.98E-07	8.04E-07	1.67E-05	0.003%	0.013%
	H	0.000163	-3.739	1.06E-09	2.74E-09	9.90E-09	9.72E-07	1.53E-08	3.34E-05	1.80E-06	2.91E-06	6.04E-05	0.010%	
CNT	C	0.009008	6.646	1.04E-07	2.69E-07	9.72E-07	9.55E-05	1.51E-06	3.28E-03	1.77E-04	2.86E-04	5.93E-03	0.977%	0.993%
	Fe	0.0001	9.45	1.65E-09	4.24E-09	1.53E-08	1.51E-06	2.38E-08	5.18E-05	2.80E-06	4.51E-06	9.36E-05	0.015%	
D-NMP	C	0.309568	6.646	3.58E-06	9.24E-06	3.34E-05	3.28E-03	5.18E-05	1.13E-01	6.09E-03	9.82E-03	2.04E-01	33.583%	98.994%
	O	0.01913	5.803	1.93E-07	4.98E-07	1.80E-06	1.77E-04	2.80E-06	6.09E-03	3.28E-04	5.30E-04	1.10E-02	1.812%	
	N	0.01913	9.36	3.12E-07	8.04E-07	2.91E-06	2.86E-04	4.51E-06	9.82E-03	5.30E-04	8.54E-04	1.77E-02	2.923%	
	D	0.55722	6.671	6.47E-06	1.67E-05	6.04E-05	5.93E-03	9.36E-05	2.04E-01	1.10E-02	1.77E-02	3.68E-01	60.676%	

$$Fractional\ Neutron\ Scatter = \frac{c_\alpha c_\beta |b_\alpha b_\beta|}{\sum c_\alpha c_\beta |b_\alpha b_\beta|} \quad \text{(Eq. 8)}$$

Supplementary Table 5. Fractional atom-pair neutron scattering contribution (Eq. 8) for 25 mg mL^{-1} SWCNTs in H-NMP. Contributions summed for each atom/species type and presented as percentage contributions. Red-yellow-blue gradient colour scale used within fractional atom-pair scattering contribution table, atom-type contribution column, and species contribution column, respectively, to provide visual aid for identifying relative scattering contributions. Neutron scattering cross sections taken from Sears[S3].

			Species	Na	Ammonia		CNT		H-NMP				Percentage Scattering Contribution	
					N	H	C	Fe	C	O	N	H		
			Atom Fraction	0.000018	0.000018	0.000163	0.009008	0.0001	0.309568	0.01913	0.01913	0.55722		
Speacies		Atom Fraction	Coherent Scattering Length (fm)	3.63	9.36	-3.739	6.646	9.45	6.646	5.803	9.36	-3.739		
Na		0.000018	3.63	2.12E-10	5.45E-10	1.97E-09	1.94E-07	3.06E-09	6.66E-06	3.59E-07	5.80E-07	6.74E-06	0.001%	0.001%
NH_3	N	0.000018	9.36	5.45E-10	1.41E-09	5.09E-09	5.00E-07	7.89E-09	1.72E-05	9.27E-07	1.49E-06	1.74E-05	0.004%	0.017%
	H	0.000163	-3.739	1.97E-09	5.09E-09	1.84E-08	1.81E-06	2.85E-08	6.21E-05	3.35E-06	5.41E-06	6.29E-05	0.014%	
CNT	C	0.009008	6.646	1.94E-07	5.00E-07	1.81E-06	1.78E-04	2.80E-06	6.10E-03	3.29E-04	5.31E-04	6.18E-03	1.333%	1.354%
	Fe	0.0001	9.45	3.06E-09	7.89E-09	2.85E-08	2.80E-06	4.42E-08	9.63E-05	5.20E-06	8.38E-06	9.75E-05	0.021%	
H-NMP	C	0.309568	6.646	6.66E-06	1.72E-05	6.21E-05	6.10E-03	9.63E-05	2.10E-01	1.13E-02	1.83E-02	2.12E-01	45.795%	98.628%
	O	0.01913	5.803	3.59E-07	9.27E-07	3.35E-06	3.29E-04	5.20E-06	1.13E-02	6.11E-04	9.85E-04	1.15E-02	2.471%	
	N	0.01913	9.36	5.80E-07	1.49E-06	5.41E-06	5.31E-04	8.38E-06	1.83E-02	9.85E-04	1.59E-03	1.85E-02	3.986%	
	H	0.55722	-3.739	6.74E-06	1.74E-05	6.29E-05	6.18E-03	9.75E-05	2.12E-01	1.15E-02	1.85E-02	2.15E-01	46.375%	

Supplementary Table 6. Fractional atom-pair neutron scattering contribution (Eq. 8) for 25 mg mL^{-1} SWCNTs in D-DMAc. Contributions summed for each atom/species type and presented as percentage contributions. Red-yellow-blue gradient colour scale used within fractional atom-pair scattering contribution table, atom-type contribution column, and species contribution column, respectively, to provide visual aid for identifying relative scattering contributions. Neutron scattering cross sections taken from Sears[S3].

			Species	Na	Ammonia		CNT		D-DMAc				Percentage Scattering Contribution	
					N	H	C	Fe	C	O	N	D		
			Atom Fraction	1.90E-05	1.90E-05	1.73E-04	9.68E-03	1.10E-04	2.64E-01	6.60E-02	6.60E-02	5.94E-01		
Species		Atom Fraction	Coherent Scattering Length (fm)	3.63	9.36	-3.739	6.646	9.45	6.646	5.803	9.36	6.671		
Na		1.90E-05	3.63	1.03E-10	2.67E-10	9.69E-10	9.64E-08	1.56E-09	2.63E-06	5.74E-07	9.26E-07	5.94E-06	0.001%	0.001%
NH_3	N	1.90E-05	9.36	2.67E-10	6.87E-10	2.50E-09	2.49E-07	4.02E-09	6.78E-06	1.48E-06	2.39E-06	1.53E-05	0.003%	0.012%
	H	1.73E-04	-3.739	9.69E-10	2.50E-09	9.09E-09	9.04E-07	1.46E-08	2.47E-05	5.38E-06	8.68E-06	5.57E-05	0.010%	
CNT	C	9.68E-03	6.646	9.64E-08	2.49E-07	9.04E-07	9.00E-05	1.45E-06	2.45E-03	5.35E-04	8.64E-04	5.54E-03	0.948%	0.964%
	Fe	1.10E-04	9.45	1.56E-09	4.02E-09	1.46E-08	1.45E-06	2.35E-08	3.96E-05	8.65E-06	1.40E-05	8.95E-05	0.015%	
D-DMAc	C	2.64E-01	6.646	2.63E-06	6.78E-06	2.47E-05	2.45E-03	3.96E-05	6.69E-02	1.46E-02	2.36E-02	1.51E-01	25.862%	99.023%
	O	6.60E-02	5.803	5.74E-07	1.48E-06	5.38E-06	5.35E-04	8.65E-06	1.46E-02	3.19E-03	5.14E-03	3.30E-02	5.645%	
	N	6.60E-02	9.36	9.26E-07	2.39E-06	8.68E-06	8.64E-04	1.40E-05	2.36E-02	5.14E-03	8.29E-03	5.32E-02	9.106%	
	D	5.94E-01	6.671	5.94E-06	1.53E-05	5.57E-05	5.54E-03	8.95E-05	1.51E-01	3.30E-02	5.32E-02	3.41E-01	58.409%	

Supplementary Table 7. Fractional atom-pair neutron scattering contribution (Eq. 8) for 25 mg mL^{-1} SWCNTs in H-DMAc. Contributions summed for each atom/species type and presented as percentage contributions. Red-yellow-blue gradient colour scale used within fractional atom-pair scattering contribution table, atom-type contribution column, and species contribution column, respectively, to provide visual aid for identifying relative scattering contributions. Neutron scattering cross sections taken from Sears[S3].

			Species	Na	Ammonia		CNT		H-DMAc				Percentage Scattering Contribution	
					N	H	C	Fe	C	O	N	H		
			Atom Fraction	1.90E-05	1.90E-05	1.73E-04	9.68E-03	1.10E-04	2.64E-01	6.60E-02	6.60E-02	5.94E-01		
Species		Atom Fraction	Coherent Scattering Length (fm)	3.63	9.36	-3.739	6.646	9.45	6.646	5.803	9.36	-3.739		
Na		1.90E-05	3.63	1.87E-10	4.82E-10	1.75E-09	1.75E-07	2.82E-09	4.76E-06	1.04E-06	1.68E-06	6.02E-06	0.001%	0.001%
NH_3	N	1.90E-05	9.36	4.82E-10	1.24E-09	4.52E-09	4.50E-07	7.27E-09	1.23E-05	2.68E-06	4.32E-06	1.55E-05	0.004%	0.016%
	H	1.73E-04	-3.739	1.75E-09	4.52E-09	1.65E-08	1.64E-06	2.64E-08	4.46E-05	9.74E-06	1.57E-05	5.65E-05	0.013%	
CNT	C	9.68E-03	6.646	1.75E-07	4.50E-07	1.64E-06	1.63E-04	2.63E-06	4.44E-03	9.69E-04	1.56E-03	5.62E-03	1.276%	1.297%
	Fe	1.10E-04	9.45	2.82E-09	7.27E-09	2.64E-08	2.63E-06	4.25E-08	7.17E-05	1.57E-05	2.53E-05	9.08E-05	0.021%	
H-DMAc	C	2.64E-01	6.646	4.76E-06	1.23E-05	4.46E-05	4.44E-03	7.17E-05	1.21E-01	2.64E-02	4.26E-02	1.53E-01	34.795%	98.686%
	O	6.60E-02	5.803	1.04E-06	2.68E-06	9.74E-06	9.69E-04	1.57E-05	2.64E-02	5.77E-03	9.31E-03	3.35E-02	7.595%	
	N	6.60E-02	9.36	1.68E-06	4.32E-06	1.57E-05	1.56E-03	2.53E-05	4.26E-02	9.31E-03	1.50E-02	5.40E-02	12.251%	
	H	5.94E-01	-3.739	6.02E-06	1.55E-05	5.65E-05	5.62E-03	9.08E-05	1.53E-01	3.35E-02	5.40E-02	1.94E-01	44.044%	

Supplementary Table 8. Fractional atom-pair X-ray scattering contribution (Eq. 9) for 25 mg mL^{-1} SWCNTs in NMP. Contributions summed for each atom/species type and presented as percentage contributions. Red-yellow-blue gradient colour scale used within fractional atom-pair scattering contribution table, atom-type contribution column, and species contribution column, respectively, to provide visual aid for identifying relative scattering contributions. Values provided for scattering at Q = 0 assuming charge-neutral atoms.

			Species	Na	Ammonia		CNT		NMP				Percentage Scattering Contribution	
					N	H	C	Fe	C	O	N	H		
			Atom Fraction	0.000018	0.000018	0.000163	0.009008	0.0001	0.309568	0.01913	0.01913	0.55722		
Species		Atom Fraction	Atomic Number (Z)	11	7	1	6	26	6	8	7	1		
Na		0.000018	11	5.15E-09	3.28E-09	4.24E-09	1.41E-06	6.76E-08	4.83E-05	3.98E-06	3.48E-06	1.45E-05	0.007%	0.007%
NH_3	N	0.000018	7	3.28E-09	2.09E-09	2.70E-09	8.95E-07	4.30E-08	3.08E-05	2.53E-06	2.22E-06	9.23E-06	0.005%	0.010%
	H	0.000163	1	4.24E-09	2.70E-09	3.49E-09	1.16E-06	5.57E-08	3.98E-05	3.28E-06	2.87E-06	1.19E-05	0.006%	
CNT	C	0.009008	6	1.41E-06	8.95E-07	1.16E-06	3.84E-04	1.85E-05	1.32E-02	1.09E-03	9.51E-04	3.96E-03	1.959%	2.053%
	Fe	0.0001	26	6.76E-08	4.30E-08	5.57E-08	1.85E-05	8.88E-07	6.35E-04	5.23E-05	4.57E-05	1.90E-04	0.094%	
NMP	C	0.309568	6	4.83E-05	3.08E-05	3.98E-05	1.32E-02	6.35E-04	4.53E-01	3.74E-02	3.27E-02	1.36E-01	67.329%	97.929%
	O	0.01913	8	3.98E-06	2.53E-06	3.28E-06	1.09E-03	5.23E-05	3.74E-02	3.08E-03	2.69E-03	1.12E-02	5.548%	
	N	0.01913	7	3.48E-06	2.22E-06	2.87E-06	9.51E-04	4.57E-05	3.27E-02	2.69E-03	2.36E-03	9.80E-03	4.854%	
	H	0.55722	1	1.45E-05	9.23E-06	1.19E-05	3.96E-03	1.90E-04	1.36E-01	1.12E-02	9.80E-03	4.08E-02	20.199%	

$$Fractional\ X\text{-}ray\ Scatter = \frac{c_\alpha c_\beta Z_\alpha Z_\beta}{\sum c_\alpha c_\beta Z_\alpha Z_\beta} \qquad \text{(Eq. 9)}$$

Supplementary Table 9. Fractional atom-pair X-ray scattering contribution (Eq. 9) for 25 mg mL^{-1} SWCNTs in DMAc. Contributions summed for each atom/species type and presented as percentage contributions. Red-yellow-blue gradient colour scale used within fractional atom-pair scattering contribution table, atom-type contribution column, and species contribution column, respectively, to provide visual aid for identifying relative scattering contributions. Values provided for scattering at Q = 0 assuming charge-neutral atoms.

			Species	Na	Ammonia		CNT		DMAc				Percentage Scattering Contribution	
					N	H	C	Fe	C	O	N	H		
			Atom Fraction	1.90E-05	1.90E-05	1.73E-04	9.68E-03	1.10E-04	2.64E-01	6.60E-02	6.60E-02	5.94E-01		
Species		Atom Fraction	Atomic Number (Z)	11	7	1	6	26	6	8	7	1		
Na		1.90E-05	11	4.19E-09	2.67E-09	3.47E-09	1.16E-06	5.73E-08	3.17E-05	1.06E-05	9.26E-06	1.19E-05	0.006%	0.006%
NH_3	N	1.90E-05	7	2.67E-09	1.70E-09	2.21E-09	7.41E-07	3.65E-08	2.02E-05	6.73E-06	5.89E-06	7.57E-06	0.004%	0.009%
	H	1.73E-04	1	3.47E-09	2.21E-09	2.87E-09	9.64E-07	4.74E-08	2.63E-05	8.76E-06	7.66E-06	9.85E-06	0.005%	
CNT	C	9.68E-03	6	1.16E-06	7.41E-07	9.64E-07	3.24E-04	1.59E-05	8.82E-03	2.94E-03	2.57E-03	3.31E-03	1.799%	1.887%
	Fe	1.10E-04	26	5.73E-08	3.65E-08	4.74E-08	1.59E-05	7.84E-07	4.34E-04	1.45E-04	1.27E-04	1.63E-04	0.089%	
DMAc	C	2.64E-01	6	3.17E-05	2.02E-05	2.63E-05	8.82E-03	4.34E-04	2.41E-01	8.02E-02	7.02E-02	9.02E-02	49.048%	98.097%
	O	6.60E-02	8	1.06E-05	6.73E-06	8.76E-06	2.94E-03	1.45E-04	8.02E-02	2.67E-02	2.34E-02	3.01E-02	16.349%	
	N	6.60E-02	7	9.26E-06	5.89E-06	7.66E-06	2.57E-03	1.27E-04	7.02E-02	2.34E-02	2.05E-02	2.63E-02	14.306%	
	H	5.94E-01	1	1.19E-05	7.57E-06	9.85E-06	3.31E-03	1.63E-04	9.02E-02	3.01E-02	2.63E-02	3.38E-02	18.393%	

Supplementary Discussion 2. Scattering of Residual Ammonia and Iron

The presence of ammonia in the sample arises from the Birch reduction route of synthesizing anionic carbon nanotubes.[S16] A fraction of ammonia is tightly bound to the metal counterion (here Na^+) and is not removed along with the bulk ammonia liquid through evaporation at reduced pressure (~ 5 mbar, room temperature). The quantity of ammonia was derived from previous CHN analysis of NaSWCNT salts which showed a 1:0.12 mol ratio of C:NH_3. The ammonia will also contribute to the neutron scattering profiles both directly and indirectly by influencing local molecules, so is therefore fit concurrently as a part of the system (assuming 100% ^{1}H and ^{14}N isotope contributions), assuming the molecules are free to dissolve in the solution (i.e. are not ion-bound when dissolved in amide). As NMP and DMAc are aprotic tertiary amides, they will not exchange hydrogens with the residual ammonia under the conditions used throughout this work.

We note that the total direct scattering contribution of ammonia is low (<0.02%, Supplementary Tables 4-7) and is not isotopically substituted here to constrain ammonia structural analysis. However, solvent molecules are well weighted and constrained by data; as both solvents show strong interaction with ammonia in the modelled system (*g(r)* CoM[NH_3]-CoM[solvent] > 2, Supplementary Figure 7), we may be confident in deriving solvent-ammonia interactions. For both solvents, the predominant interaction is N-H⋯O bonding, with peaks at 2.10 Å (NMP) and 2.04 Å (DMAc) (Supplementary Figure 7c) which shows minimal directionality with regards to the oxygen

(Supplementary Figure 7d). For NMP, this is the only significant motif, while DMAc shows a fraction of ordered N···H-C bifurcated with the trans-oxygen (Me^Z) and acetyl (Me^A) methyls, seen as minor *g(r)* N(NH_3)-C(DMAc) peaks around 5.3 Å (Supplementary Figure 7bii) and the lobes in the SDF which for a crescent at higher sampled percentages (Supplementary Figure 7dii). While the solvation shell around ammonia is well defined, the ordering diminishes quickly and the second shell is substantially less pronounced for both NMP and DMAc.

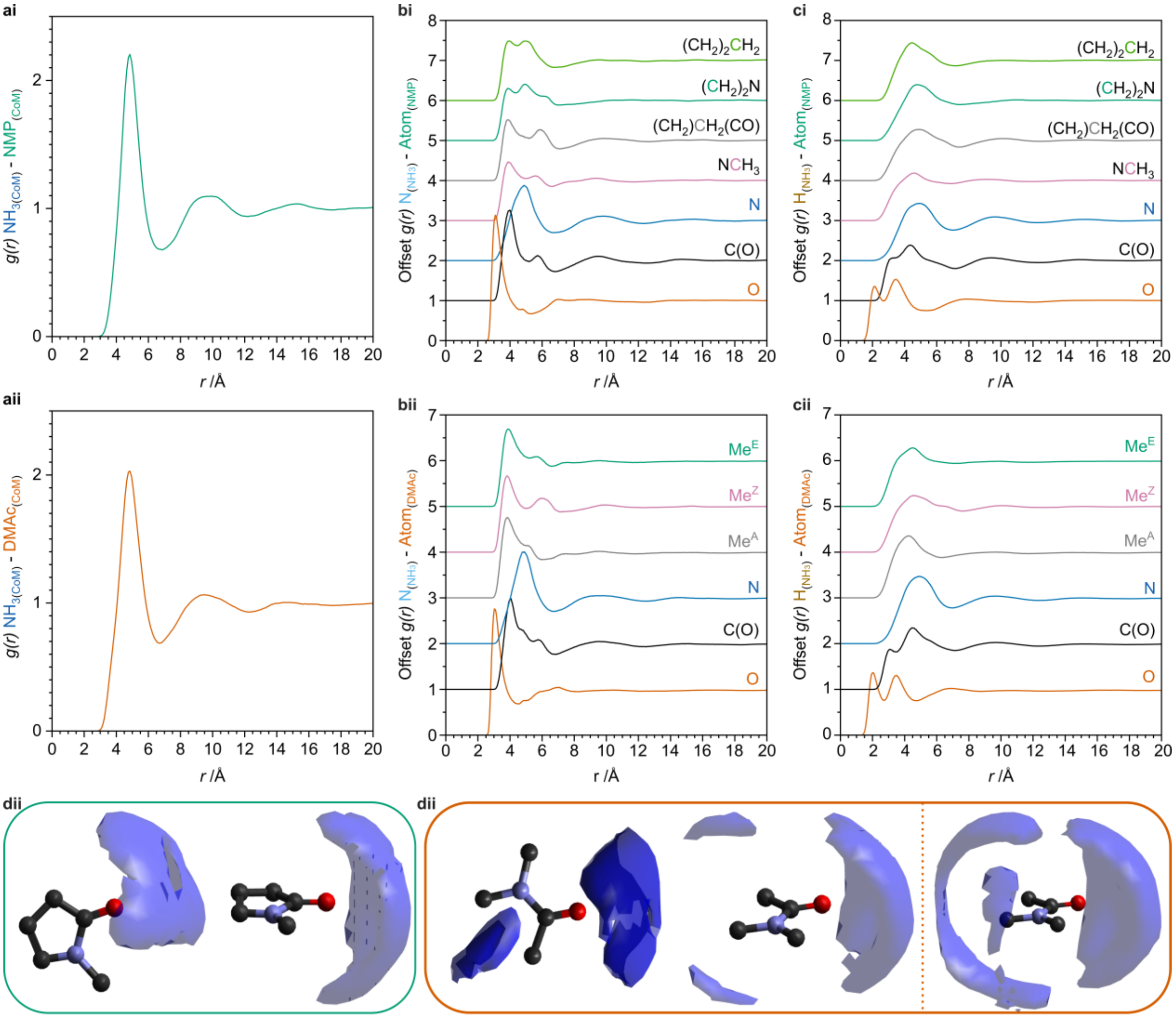

Supplementary Figure 7. **Residual ammonia-solvent structure**. (a) CoM-CoM radial distribution function of ammonia and (i) NMP and (ii) NMP. (b-c) Atom-atom radial distribution functions of ammonia's (b) nitrogen and (c) hydrogen, as a function of distance from each constituent framework atom of (i) NMP and (ii) DMAc, with graphs y-offset in multiples of 1. (d) SDFs of ammonia CoM around (i) NMP and (ii) DMAc (C black; N blue; O red; H omitted for clarity) for 10% most likely ammonia positions at two viewing angles and (right of dashed line) 20% most likely position around DMAc with one viewing angle to show crescent feature bifurcating Me^A/Me^Z.

The ordering of ammonia around CNTs is significantly less well constrained than solvent-based structuring in our model as no isotopic substitution of CNT or NH_3 was undertaken, and both fractions are weak contributors to the total direct scattering signal

(NH_3 <0.02%, CNT <1.5%, Supplementary Tables 4-7) and these results have lower confidence than the solvent-based analyses. A fraction of the co-solvated ammonia molecules lie close to the charged SWCNT surface for both solvents (Supplementary Figure 8a) with RDFs showing hydrogen atoms that point towards the surface (Supplementary Fig. 8b,c), similarly to the arrangement found in anionic fullerene ammonia solutions.[S9] However, while the hydrogen and nitrogen peaks in the *g(r)*s of fulleride and ammonia are distinct and separated by 1 Å, here the two peaks are closer and less defined indicating that more than one orientation is present in solution. These proximally located molecules constitute only a small fraction of the total ammonia (3%). The remaining NH_3 species tend to show higher concentrations in the center of solvation shells for DMAc (possibly due to good solvation combined with the higher DMAc densities in the shell centers) but are otherwise randomly distributed.

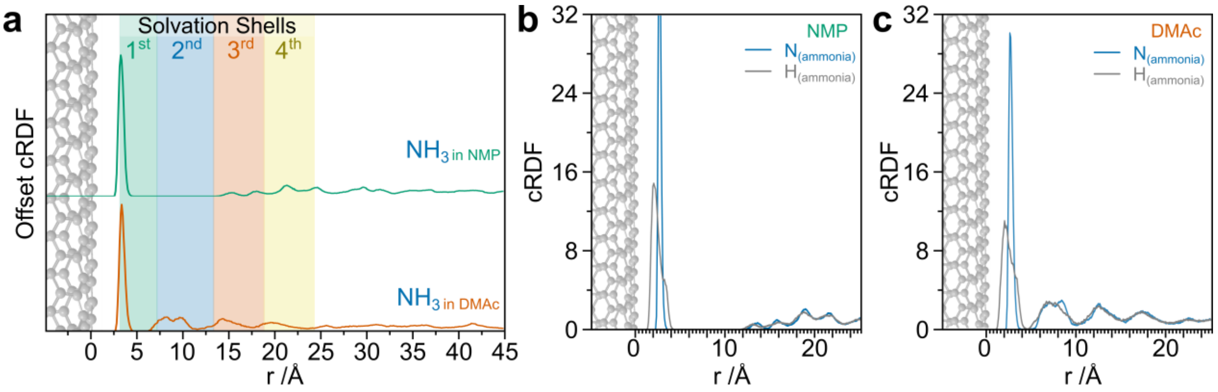

Supplementary Figure 8. **Residual ammonia structure in solution**. (a) CoM cylindrical Radial distribution functions of ammonia showing correlations to the carbon nanotube central axis (distance offset by CNT radius). (b,c) Atomic cRDFs of N (blue) and H (grey) of ammonia in NMP and DMAc solutions, respectively.

Iron is present in many single-walled nanotubes as a residue from the nanotube synthesis (including the HiPCO process used here [S17]). The majority of iron can be removed via washing in HCl, and samples purchased here have undergone this treatment, however a fraction (~5 wt%) remains present encapsulated in the ends of nanotubes. Removal of all iron is possible but requires aggressive oxidation chemistry to degrade the higher curvature nanotube ends to expose the iron for acidic dissolution.[S18] Unfortunately, it is well established that these oxidation chemistries unavoidably also introduce defects into the nanotube sidewall which would locally disrupt solvent-nanotube structure. In contrast, the encapsulated iron has no solvent interface and would therefore not be expected to significantly affect the solvent behaviour. The total neutron scattering contribution from the iron ranges between 0.015% – 0.021% (Supplementary Tables 4-7) and is therefore omitted in the analysis performed here.

Supplementary Discussion 3. Classical Monte Carlo simulation of Na-SWCNT salt in a Dielectric Medium

To better understand the impact of using a fully molecular liquid model of a charged surface on the counterion distribution, it is useful to compare the EPSR derived systems to a classic field-based model where the solvent is represented by a universal dielectric field. For consistency with the (Monte Carlo-based) EPSR experimentally guided model, classical Monte Carlo (MC) modelling is the obvious approach. Charged interfaces have been studied extensively with MC [S19] and have been applied to understand interactions and aggregation phenomena in nanoparticles [S20,S21].

Classical MC simulations were conducted for a negatively charged (7,6) SWCNT and charge-balancing sodium ions embedded in a dielectric medium, using the program, *Monte*[S22,S23]. To elucidate trends, these simulations were conducted for the following values of relative permittivity, ε_r: 1, 8 (tetrahydrofuran), 20, 33 (**NMP**), 38 (**DMAc**), 47 (dimethylsulfoxide), 60, 80 (water), 120, 180 (N-methylformamide, NMF). The simulation cell contained a 96 Å length of negatively charged (7,6) chirality SWCNT and 84 charge-balancing Na^+ cations. For reference, the SWCNT has a cylindrical radius of 4.47 Å and the hexagonal unit cell had the nanotube lying along the x-axis and unit cell vectors:

$$\begin{pmatrix} 96.0156 & 0.0 & 0.0 \\ 0.0 & 90.0 & 0.0 \\ 0.0 & 45.0 & 77.9423 \end{pmatrix} \text{Å}.$$

A real-space cut-off of 12 Å was applied along with Ewald summation of long-range Coulombic interactions using a reciprocal-space cut-off of 2.3 $Å^{-1}$ and convergence factor α = 0.24. Simulations were conducted at 300 K within an NVT ensemble. The starting configuration placed Na^+ ions at random within the system, but at least 20 Å from the SWCNT central axis (i.e., >15 Å from the SWCNT wall). Equilibration was conducted over approximately 10,000,000 MC steps, by which point structure and internal energy were fluctuating around constant values. Sampling took place over approximately a further 10,000,000 steps.

A combination of Lennard-Jones (LJ) and Coulomb potentials were used to represent interaction potentials, $U_{\alpha\beta}(r)$, between atom pairs α and β in the system:

$$U_{\alpha\beta}(r) = 4\varepsilon_{\alpha\beta}\left[\left(\frac{\sigma_{\alpha\beta}}{r}\right)^{12} - \left(\frac{\sigma_{\alpha\beta}}{r}\right)^{6}\right] + \frac{1}{4\pi\varepsilon_0\,\varepsilon_r}\frac{q_\alpha q_\beta}{r} \qquad \text{(Eq. 8)}$$

where $\varepsilon_{\alpha\beta}$ and $\sigma_{\alpha\beta}$ are as given in Supplementary Table 1 with Lorentz-Berthelot mixing rules[S10] applied for the cross terms, ε_0 is the permittivity of free space and ε_r the relative permittivity. To analyze the data, we measure the cumulative fraction of sodium cations (N) with respect to the charged SWCNT central axis, as a function of relative permittivity.

The models show a segregation of behaviors between surface absorbed sodium ions and sodium ions at further distances. The distance between condensed ions and the surface increased as a function of permittivity (Supplementary Figure 9) but had virtually

stabilized by 9 Å from the central SWCNT axis in all cases, which is taken as the condensed-dissociated threshold for all modelled systems (Supplementary Table 10). The condensed layer also showed a greater distribution of SWCNT-Na distances within the condensed layer with increasing permittivity as seen in snapshots of the system (Supplementary Figure 10).

As permittivity increases, the shielding of the surface charge facilitated a greater degree of dissociation. However, in stark contrast to the experimentally guided system discussed in the main text, here, the vast majority of the counterions condensed on the nanotube surface in all cases. The DMAc-matched dielectric (ε_r = 38) lead to 98.9% of the ions condensing, versus 17% seen in experiment modelled by EPSR. Even at an extremely high permittivity such as 180 (approximately that of NMF and over twice that of water's 80.1 at 20 °C), over 85% of the ions are still condensed. These results highlight the importance of treating the solvent as discrete molecules in contrast to a simple dielectric continuum.

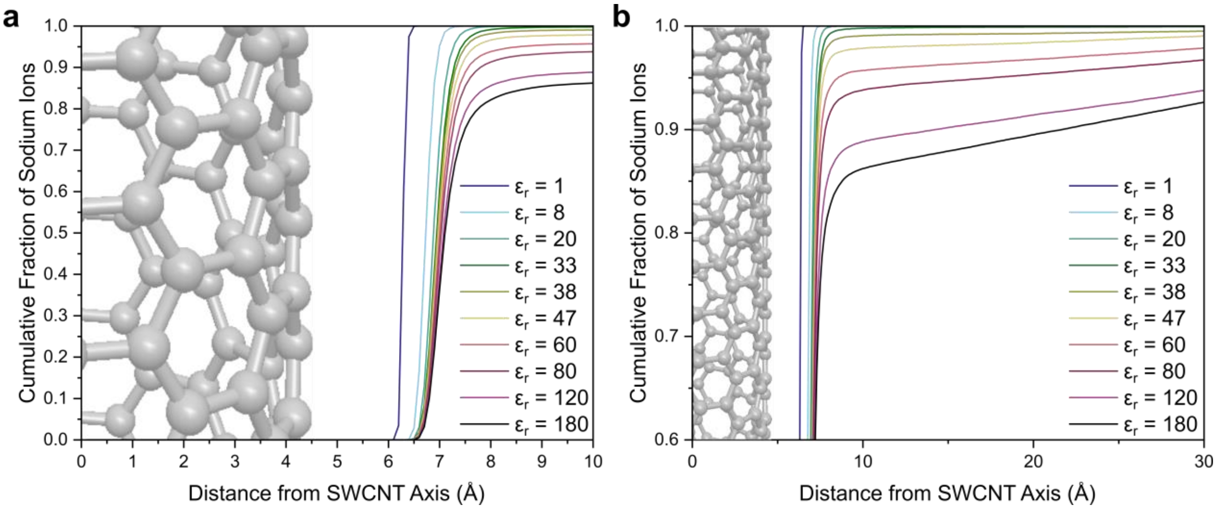

Supplementary Figure 9. Cumulative distribution of sodium ions as a function of distance from the SWCNT central axis for systems modelled with different relative permittivity. (a) Short range to highlight differences in condensed ions, (b) at larger distances to highlight dissociated ion distributions. Schematic (7,6) nanotube overlain to illustrate distance from SWCNT wall.

Supplementary Table 10. Fraction of sodium atoms within 9 Å of the central SWCNT axis determined by classical Monte Carlo over a range of relative permittivity.

ε_r	N(≤9Å)/N(total)
1	1
8	1
20	1
33	0.997
38	0.989
47	0.977
60	0.954
80	0.933
120	0.882
180	0.854

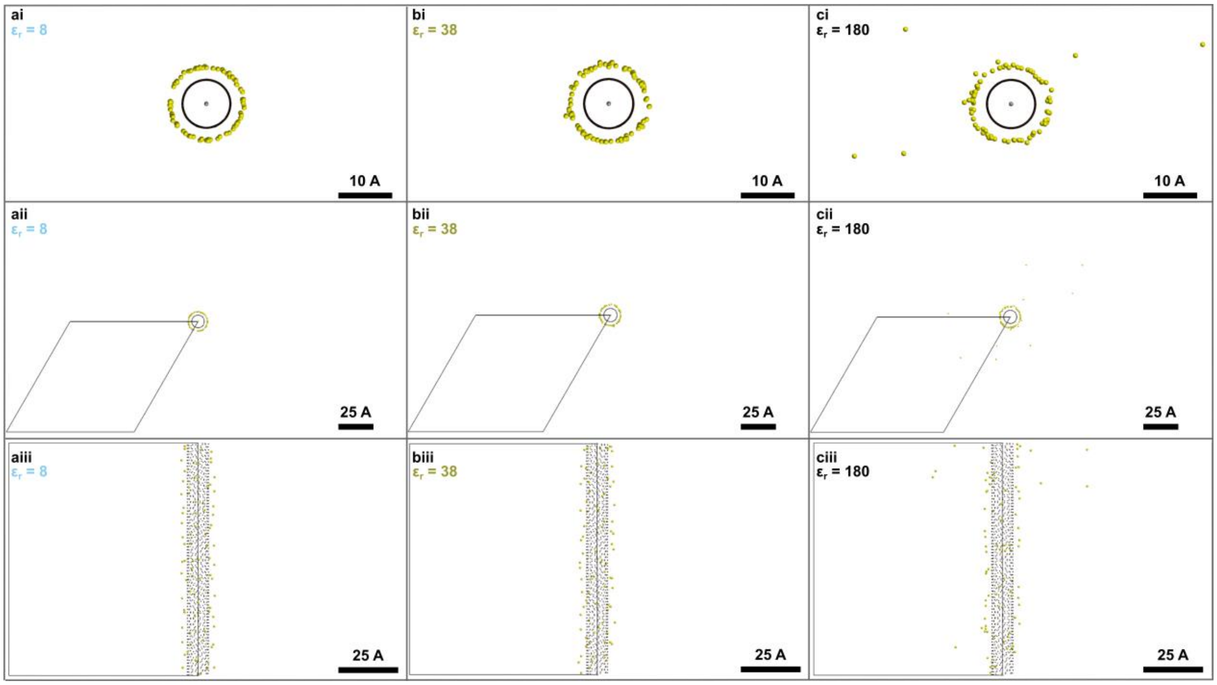

Supplementary Figure 10. Snapshots of Monte Carlo. Modelled at relative permitivities of (a) 8, (b) 38 – equal to DMAc, and (c) 180. Models shown down (i, ii) SWCNT axis and (iii) perpendicular to SWCNT axis. Black, carbon; yellow, sodium; grey, null atoms used to highlight SWCNT central axis. Black outline highlights unit cell dimensions of initial SWCNT model.

Supplementary Discussion 4. Comparison of Model to X-Ray Scattering

Similar to neutrons, X-rays of suitable wavelength may be used for liquid scattering to derive structural information. This approach is particularly useful when the samples contain atoms of disparate atomic number (Z) as the atomic/ionic X-ray scattering factors scale linearly with the number of electrons (at $Q = 0$). In principle it is possible to integrate X-ray with neutron scattering data as an additional fitting constraint in EPSR which allows concurrent fitting with neutron scattering data of isotopologues. However, in practice this integration comes with significant complicating factors. These include mismatched X-ray and neutron instrument Q-ranges, and inherent lack of consistency in the data correction and reduction procedures for the two probes. Notably the *Q*-dependence of X-ray atomic form factors, challenges in absolute normalisation of the X-ray scattering signal (there is no equivalent to the vanadium normalisation method of neutrons), and concomitant need to account for X-ray Compton scattering.

These challenges risk introducing unphysical artefacts into the analysis and we must therefore rationalize on a case-by-case basis whether the inclusion of X-ray data as an additional EPSR constraining dataset will in fact improve the reliability of the final fitted model. In the solutions measured in this work, the solvent is the dominant X-ray scattering component (~98% of the total scattering, Supplementary Tables 8-9), providing similar information to the neutron datasets. Notably, sodium's direct scattering contribution (0.007%) is small due to its low-Z and low concentration. In contrast, the high-Z of the residual iron (see Supplementary Discussion 2) becomes a measurable

contributing factor that cannot be accounted for in the EPSR model cell, and which is therefore highly likely to introduce compensatory artefacts into the analysis cell.

While we do not, therefore, believe that inclusion of X-ray scattering data as a fitting constraint is appropriate in the current case, it does provide an opportunity to cross-validate the neutron-based EPSR solvent structure refinement against an independent, solvent-specific probe. For this reason, X-ray diffraction measurements were performed on a Malvern Panalytical Empyrean using a silver anode (λ = 0.5594 Å, 60 kV). Samples were loaded in a glovebox in a borosilicate glass capillary (Capillary Tube Supplies Ltd., 2 mm outer diameter, 0.01 mm wall) and sealed with wax (Hawksley) and mounted on a spinning capillary holder. Measurements were taken between 2θ = 3° – 145.8° at 0.1° increments over 1 h, and cycled for 24 iterations. Data was processed using GudrunX.[S1]

The X-ray scattering data shows good agreement with the EPSR-derived structures in both reciprocal (Supplementary Figure 11b) and real (Supplementary Figure 11c) space with peak centers consistent throughout. This highlights that the structures derived through EPSR fitting to neutron datasets are consistent with X-ray scattering. No additional features are seen in the experimental data. Peak intensities are mismatched in places, with a systematic offset seen in the DMAc $G(r)$ in particular, which is likely attributable to the aforementioned data correction and background subtraction. Compared to the neutron scattering datasets, the difference between X-ray scatter of

solvent and nanotube solutions have significantly smaller differences (Supplementary Figure 12, Supplementary Figure 3-5, Extended Data Figures 1-2), attributed to the hydrogen insensitivity of X-rays and smaller accessible Q-range versus the NIMROD instrument (which was specifically designed for the structure of disordered materials across multiple length scales).

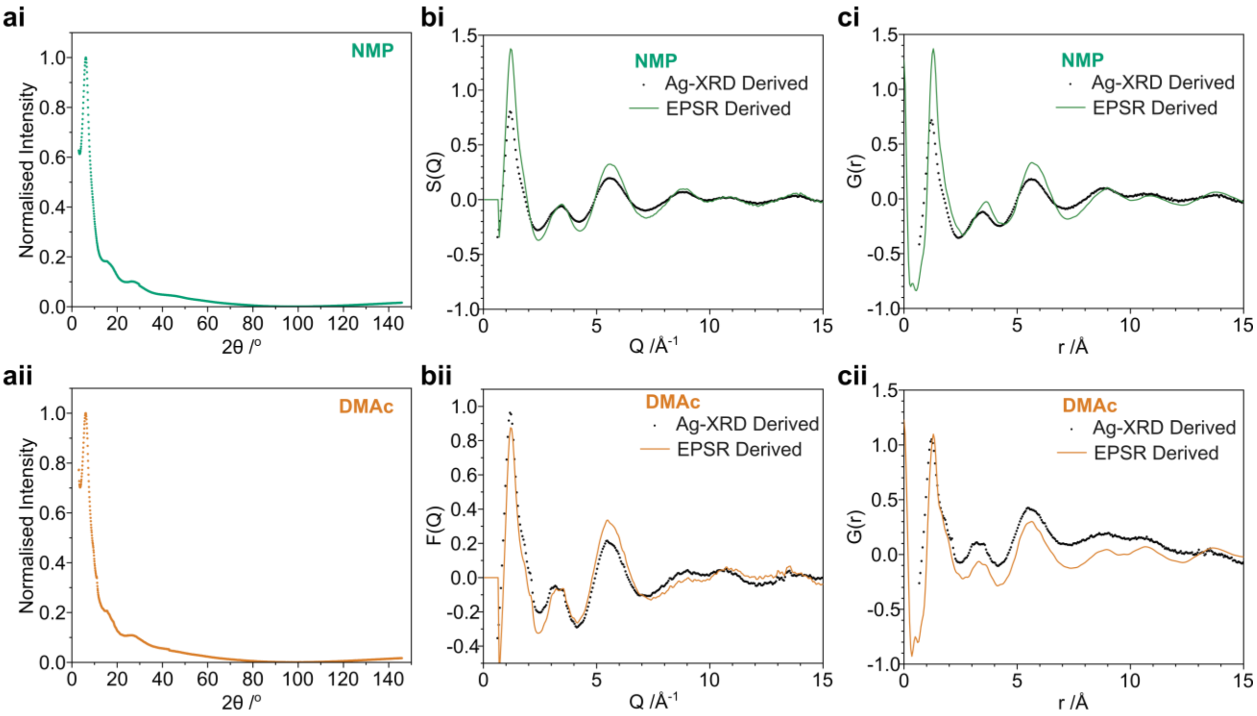

Supplementary Figure 11. Ag-Source X-ray scattering versus neutron-EPSR derived model. (a) Normalised X-ray scattering data. (b) Total structure factors. (c) Composite radial distribution functions, each for (i) NMP and (ii) DMAc solutions of NaSWCNT.

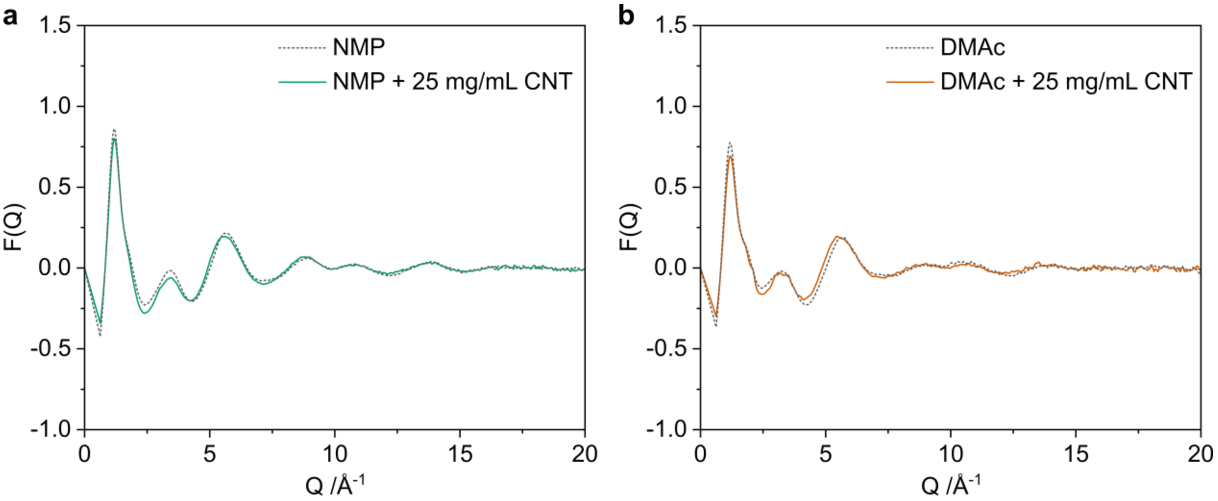

Supplementary Figure 12. Pure solvent versus nanotubide solutions. F(Q) from corrected TXRS data for solvent and 25 mg mL^{-1} NaSWCNT solutions for (a) NMP and (b) DMAc.

References

S1 Soper, A. K. *GudrunN and GudrunX: programs for correcting raw neutron and X-ray diffraction data to differential scattering cross section*. (Science & Technology Facilities Council, 2011).

S2 Soper, A., Howells, W. & Hannon, A. Analysis of Time-of-Flight Diffraction Data from Liquid and Amorphous Samples. *ISIS Facility, RAL, Oxon, UK* **11** (1989).

S3 Sears, V. F. Neutron scattering lengths and cross sections. *Neutron News* **3**, 26-37, doi:10.1080/10448639208218770 (1992).

S4 Soper, A. K. Inelasticity corrections for time-of-flight and fixed wavelength neutron diffraction experiments. *Mol. Phys.* **107**, 1667-1684 (2009).

S5 Soper, A. Empirical Potential Monte Carlo Simulation of Fluid Structure. *Chem. Phys.* **202**, 295-306 (1996).

S6 Thompson, H. *et al.* The structure of polaronic electron cavities in lithium–ammonia solutions. *J. Phys. Condens. Matter* **16**, 5639 (2004).

S7 Bowron, D. *et al.* NIMROD: The Near and InterMediate Range Order Diffractometer of the ISIS Second Target Station. *Rev. Sci. Instrum.* **81**, 033905 (2010).

S8 Howard, C. A. & Skipper, N. T. Computer simulations of fulleride anions in metal-ammonia solutions. *J. Phys. Chem. B* **113**, 3324-3332 (2009).

S9 Howard, C. A., Thompson, H., Wasse, J. C. & Skipper, N. T. Formation of Giant Solvation Shells Around Fulleride Anions in Liquid Ammonia. *J. Am. Chem. Soc.* **126**, 13228-13229 (2004).

S10 Lorentz, H. A. Ueber die Anwendung des Satzes vom Virial in der kinetischen Theorie der Gase. *Ann Phys* **248**, 127-136 (1881).

S11 Jorgensen, W. L., Maxwell, D. S. & Tirado-Rives, J. Development and testing of the OPLS all-atom force field on conformational energetics and properties of organic liquids. *J. Am. Chem. Soc.* **118**, 11225-11236 (1996).

S12 Jorgensen, W. L. & Swenson, C. J. Optimized intermolecular potential functions for amides and peptides. Structure and properties of liquid amides. *J. Am. Chem. Soc.* **107**, 569-578 (1985).

S13 Chen, Y.-G. & Weeks, J. D. Local molecular field theory for effective attractions between like charged objects in systems with strong Coulomb interactions. *Proceedings of the National Academy of Sciences* **103**, 7560-7565 (2006).

S14 Youngs, T. G. Aten 2.1.9. *GitHub Repository*, https://github.com/trisyoungs/aten/releases/ (2018).

S15 Youngs, T. G. dlputils. *GitHub Repository*, https://github.com/trisyoungs/dlputils (2023).

S16 Fogden, S. a., Howard, C. A., Heenan, R. K., Skipper, N. T. & Shaffer, M. S. Scalable Method for the Reductive Dissolution, Purification, and Separation of Single-Walled Carbon Nanotubes. *ACS nano* **6**, 54-62 (2011).

S17 Nikolaev, P. Gas-phase production of single-walled carbon nanotubes from carbon monoxide: a review of the HiPco process. *Journal of Nanoscience and Nanotechnology* **4**, 307-316 (2004).

S18 Clancy, A. J., White, E. R., Tay, H. H., Yau, H. C. & Shaffer, M. S. Systematic comparison of conventional and reductive single-walled carbon nanotube purifications. *Carbon* **108**, 423-432 (2016).

S19 Henderson*, D., Gillespie, D., Nagy, T. & Boda, D. Monte Carlo simulation of the electric double layer: dielectric boundaries and the effects of induced charge. *Molecular Physics* **103**, 2851-2861 (2005).

S20 Liu, H. H., Lanphere, J., Walker, S. & Cohen, Y. Effect of hydration repulsion on nanoparticle agglomeration evaluated via a constant number Monte–Carlo simulation. *Nanotechnology* **26**, 045708 (2015).

S21 Paula, F. *et al.* pH-dependent phase transitions in ferrofluids: A Monte Carlo simulation study using an extended DLVO model. *Colloids Surf. A Physicochem. Eng. Asp.* **658**, 130578 (2023).

S22 Skipper, N. T. *MONTE: User's Manual: Technical Report*. (Department of Chemistry, University of Cambridge, 1992).

S23 Skipper, N., Sposito, G. & Chang, F.-R. C. Monte Carlo simulation of interlayer molecular structure in swelling clay minerals. 2. Monolayer hydrates. *Clays Clay Miner.* **43**, 294-303 (1995).
